# Congruency and distance effects vary across simultaneous and sequential two-digit integer, fraction, and decimal 2AFC tasks

**DOI:** 10.3758/s13428-026-02958-6

**Published:** 2026-04-13

**Authors:** Isabella Starling-Alves, Eric D. Wilkey

**Affiliations:** 1 Department of Psychology and Human Development (Peabody College), Vanderbilt University, 230 Appleton Place; PMB 552, Nashville, TN 37203 USA; 2 Department of Educational Psychology, University of Nebraska Lincoln, Lincoln, NE USA

**Keywords:** 2AFC, Alternative forced choice, Simultaneous comparison, Sequential comparison, Distance effect, Congruency effect, Number

## Abstract

Two-alternative forced choice (2AFC) comparison tasks are widely used to investigate how humans process complex information. However, it is still not fully understood how the format of these tasks influences cognitive strategies. In this study, we analyzed whether 2AFC tasks with simultaneous stimulus presentation provide the same information on how people process information as 2AFC tasks with sequential stimulus presentation. We investigated this question within the number domain. In particular, we investigated how the distance and congruency effects—which are interpreted as behavioral markers of holistic and fragmented number processing—vary across simultaneous and sequential versions of two-digit integer, fraction, and decimal 2AFC tasks. Undergraduate students (*n* = 162) completed both simultaneous and sequential versions of two-digit integer, fraction, and decimal comparison tasks. Comparison pairs varied in numerical distance (near vs. far) and congruency (congruent vs. incongruent). Across all number types, we observed both distance and congruency effects. However, these effects differed by task format. In particular, a linear mixed model revealed that the congruency effect was stronger in simultaneous tasks with near-distance pairs and weaker in sequential tasks with far-distance pairs. The differences in the strength of the distance and congruency effects across simultaneous and sequential 2AFC task formats indicate that they play a critical role in shaping information processing strategies. Thus, our findings suggest that simultaneous and sequential 2AFC tasks engage distinct cognitive processes and should not be used interchangeably in cognitive research.

**Supplementary Information:**

The online version contains supplementary material available at 10.3758/s13428-026-02958-6.

Understanding how people process information and make decisions is a central question in cognitive psychology. To address this question, researchers often rely on experimental paradigms such as the two-alternative forced-choice (2AFC) task. In 2AFC tasks, participants are presented with two stimuli and must choose between them (Sparks et al., 2022). This experimental paradigm has been widely used to investigate a range of cognitive functions, including perceptual discrimination, lexical decision, and number processing (Rattat & Chevalier, 2020; Ratcliff et al., 2018; Rogers et al., 2004; Wilkey et al., 2020; Yeshurun et al., 2008). In these studies, participants’ performance is usually analyzed in terms of accuracy and response time, which serve as proxies for identifying differences across stimuli and making decisions about them.

The 2AFC tasks were originally developed to measure perceptual sensitivity, particularly to determine the smallest detectable difference between stimuli using psychophysics (Fechner et al., 1948). This experimental paradigm has become popular in cognitive science because it offers several methodological strengths. For instance, since participants are encouraged to provide a response, they must discriminate between the two options and engage in a decision-making process (Thurstone, 1927). This setup promotes binary, discrimination-based decisions within a controlled stimulus space, providing unbiased information when an appropriate number of trials is used, while maintaining time efficiency and lower cognitive load compared to tasks with a greater number of alternatives (Vancleef et al., 2018). Furthermore, 2AFC tasks allow for the simultaneous modeling of accuracy and response time, combining these performance metrics into cohesive evidence-accumulation models (Ratcliff et al., 2018). Thus, 2AFC tasks offer a reliable, efficient way to assess information processing and decision-making with low cognitive demands.

Despite the strengths of the 2AFC paradigm, its format may influence conclusions about the cognitive mechanisms supporting perceptual and decision-making processes (Bogacz et al., 2006; García-Pérez & Alcalá-Quintana, 2020). For example, participants’ accuracy in 2AFC tasks tends to decrease when they are required to discriminate between two stimuli presented sequentially (i.e., one after the other) compared to simultaneously (i.e., side by side) (Mou et al., 2024). This difference may be due to different cognitive heuristics elicited by simultaneous and sequential tasks. In a sequential presentation task, participants may analyze each stimulus holistically, integrating all available information and then recruiting the relevant ones during the decision-making process. In contrast, in a simultaneous presentation task, participants may engage in feature-by-feature comparisons, focusing on the most salient differences between isolated components of the stimuli (Basu & Savani, 2019; Hsee & Leclerc, 1998). Thus, sequential 2AFC tasks tend to elicit more holistic processes, whereas simultaneous 2AFC tasks tend to elicit more fragmented processing. Understanding these differences is critical, since the validity of certain findings in cognitive psychology may depend on the specific experimental paradigm used.

In the present study, we investigate how 2AFC task format (sequential vs. simultaneous) influences conclusions about information processing using multicomponent numbers (i.e., two-digit integers, fractions, and decimals). Multicomponent numbers are those composed of more than one numerical element or digit. In everyday life, we frequently encounter such numbers. For example, time is often represented by multi-digit integers (e.g., 45 min), quantities in recipes are commonly expressed with fractions (e.g., 3/4 cup of sugar), and prices are typically written as decimals (e.g., $4.65). It has been debated whether people process the absolute magnitude of these numbers in an integrated way or process each component separately (Bonato et al., 2007; Dehaene et al., 1990; Nuerk & Willmes, 2005; Toomarian & Hubbard, 2018). Thus, these numbers serve as an effective model for investigating how the 2AFC format influences holistic versus fragmented information processing.

## Modeling the effects of 2AFC task format through multicomponent number processing

Traditionally, when 2AFC comparison tasks are used to model multicomponent number processing, participants are presented with two numbers and asked to indicate which one is greater. In these tasks, the presence of the *distance effect* (slower and less accurate comparisons when numbers are near each other on a number line) has been used as a marker of holistic number processing (Dehaene et al., 1990; Toomarian & Hubbard, 2018). In contrast, the presence of the *congruency effect* (slower and less accurate comparisons when the magnitude of individual number parts conflict with the holistic magnitude) has been used as a marker of fragmented processing (Nuerk et al., 2001; 2004). In this study, we examined how simultaneous and sequential 2AFC tasks elicit these two effects in two-digit integer, fraction, and decimal comparisons to understand the impact of task format on information processing. In the following, we briefly review how the distance and congruency effects inform study conclusions about holistic and fragmented information processing, and how they may change as a function of contextual factors, including task format.

### **Distance effect**

The distance effect is one of the most well-documented phenomena in numerical cognition research. This effect refers to the finding that people are faster and more accurate in comparing numbers separated by a far distance (e.g., 1 vs. 9, distance = 8) than by a near distance (e.g., 1 vs. 2, distance = 1; Moyer & Landauer, 1967). The distance effect is elicited in tasks involving nonsymbolic numerical magnitudes (e.g., comparing the number of two sets of dots; Lonnemann et al., 2011; Piazza et al., 2004) and symbolic numbers, including numerals or number words representing single-digit integers (Cao et al., 2010; Dehaene, 1996; Lukas et al., 2014). The robustness of the distance effect across development (Holloway & Ansari, 2009) and number formats (Matthews & Chesney, 2015; Smets et al., 2013), and even the fact that it is phylogenetically preserved across many species (Nieder, 2020), suggests that it reflects fundamental properties of numerical magnitude processing in the human brain (Dehaene, 2003).

While the distance effect has been extensively observed in single-digit number tasks (Hubbard et al., 2005), its manifestation in multicomponent numbers remains less clear. Multicomponent numbers introduce additional structural features (e.g., place value in two-digit integers and decimals; numerators and denominators in fractions), which may be processed differently from single-digit numbers. Examining how the distance effect manifests in multicomponent number comparisons can provide insight into whether they are processed holistically or in a fragmented manner.

Studies investigating the distance effect in multicomponent numbers have yielded conflicting results. For example, in the case of two-digit integers, Dehaene and colleagues (1990) found that adults performed better when comparing two-digit integers separated by a far absolute distance (e.g., 65 vs. 51, distance = 14) than a near-absolute distance (e.g., 65 vs. 59, distance = 6). This observed distance effect suggests that adults can process the magnitude of two-digit integers holistically. However, other studies have shown that adults’ performance in two-digit integer comparisons is influenced not only by the absolute distance between the numbers (e.g., 65 vs. 51, distance = 14) but also by the distance between their isolated components, including both units (e.g., 65 vs. 51, unit distance = 4) and decades (e.g., 65 vs. 51, decade distance = 1; Nuerk et al., 2001, 2004). These findings suggest a *hybrid* strategy that combines fragmented and holistic processing.

Similar patterns have been observed for fractions. Toomarian and Hubbard (2018) found that adults exhibited a distance effect based on the absolute distance between two fractions (e.g., 1/2 vs. 5/9, absolute distance = 0.06), suggesting that they can directly access the *holistic* magnitude of these numbers. In contrast, Bonato and colleagues (2007) found that the distance effect is modulated by differences between denominators (e.g., 1/5 vs. 1/9, denominator distance = 4) but not by the absolute difference between the fractions. These findings indicate that people process multicomponent numbers in a *fragmented* way: they might initially process only parts of the number separately, instead of integrating numerators and denominators into a holistic magnitude. Other studies have found that adults compare pairs of fractions based on both their holistic magnitudes and the magnitude of their numerators and denominators alone (DeWolf & Vosniadou, 2015; Van Hoof et al., 2020), suggesting that they engage in a hybrid strategy.

Finally, there is also mixed evidence on how decimal comparisons elicit the distance effect. For instance, a computational model found that decimal number processing is better explained by representations of each digit separately rather than holistic magnitudes (Huber et al., 2016), suggesting fragmented processing. However, other studies have found that decimal comparisons also elicit a distance effect based on the absolute magnitude of the numbers (Cohen, 2010; Rosenberg-Lee et al., 2023), supporting a hybrid approach. To better understand how these different accounts explain multicomponent number processing across different contexts, we compared the distance effect in simultaneous and sequential two-digit integer, fraction, and decimal comparison tasks.

### **Congruency effect**

Beyond the distance effect, another way to investigate how people process multicomponent numbers is by examining the congruency effect. This effect occurs when people are faster and more accurate when comparing numbers where holistic magnitudes and component-based magnitudes align (i.e., congruent trials) than numbers where the component-based magnitudes conflict with the overall holistic magnitude (i.e., incongruent trials). This effect suggests that the components of multicomponent numbers are processed separately and can interfere with a direct access to the holistic magnitude.

The congruency effect may arise from multiple factors. One possibility is that it reflects people primarily processing multicomponent numbers in a fragmented rather than a holistic way. For example, when comparing 83 versus 29, participants may automatically process the components separately, such as the decades “8” and “2” and the units “3” and “9.” If they compare only the unit digits rather than the holistic magnitudes, they might erroneously indicate that 29 is greater than 83, since 9 is greater than 3. This fragmented comparison can impair performance on number pairs where the fragmented and holistic magnitudes are misaligned.

An alternative explanation is related to efficiency in engaging in holistic and componential strategies (Evans, 2008; Graziano, 2018; Van Hoof et al., 2020). People may directly process the holistic magnitudes of multicomponent numbers, but often focus on their individual components due to extensive experience with single-digit numbers. In such cases, executive resources may be needed to inhibit automatic fragmented processing in favor of holistic strategies (Rosenberg-Lee et al., 2023). For instance, when comparing 1/2 versus 3/8 in a fraction task, participants may access their holistic magnitudes but automatically process “1,” “2,” “3,” and “8” separately due to their familiarity with single-digit numbers acquired through schooling and everyday experiences. Participants must inhibit the fragmented representations in order to engage in holistic number processing (e.g., inhibit the representation of magnitudes “1” and “3” to process the absolute magnitudes “1/2” and “3/8”). However, this inhibitory mechanism is only necessary for number pairs in which the fragmented and holistic magnitudes conflict (in the example, 1/2 is greater than 3/8, but 1 < 3 and 2 < 8). The recruitment of inhibitory mechanisms in incongruent trials may make them slower and less accurate than congruent trials, leading to a congruency effect.

While these accounts differ in assumptions related to mental representation of multicomponent numbers (i.e., fragmented vs. hybrid processing), they converge in emphasizing the automatic processing of single-digit components and the critical role of attentional and executive resources in multicomponent number processing (Wilkey, 2023). In particular, both accounts acknowledge that multicomponent number processing is cognitively demanding, requiring either working memory resources to integrate individual number parts, or inhibitory control to suppress fragmented processing in favor of holistic processing.

The congruency effect has been observed in two-digit integer (Artemenko et al., 2024; Bahnmueller et al., 2021; Nuerk et al., 2001; Wood et al., 2006), fraction (e.g., 3/8 is greater than 1/2 because 3 is greater than 1 and 8 is greater than 2; Gómez et al., 2015; Obersteiner et al., 2013), and decimal comparison tasks (e.g., 0.39 vs. 0.52, where 0.39 < 0.52 but 9 > 5; Rosenberg-Lee et al., 2023; Varma & Karl, 2013), suggesting that these different number types may be processed in a fragmented way. However, as discussed above, the distance effect has also been observed in 2AFC tasks using these number types as stimuli. Thus, it is possible that whether people engage in fragmented or holistic processing in 2AFC multicomponent number comparisons depends on contextual factors.

## How 2AFC task format may influence distance and congruency effects

The investigation of multicomponent number processing has frequently relied on 2AFC comparison tasks, implemented in simultaneous and sequential formats (Kuzmina et al., 2023; Price et al., 2012; Smets et al., 2016; Schneider et al., 2017; Schwenk et al., 2017). In the literature, these two task formats have been used interchangeably to investigate underlying mechanisms of number processing, including the distance and the congruency effects (e.g., DeWolf et al., 2016; Ischebeck et al., 2010). However, even though these task formats are generally assumed to measure the same underlying construct, some studies suggest that different task formats elicit different processing strategies.

Some studies have found that the format of 2AFC tasks influences information processing when numerical stimuli are used (e.g., Ganor-Stern et al., 2009; Zhang & Wang, 2005). For example, Ganor-Stern and colleagues (2009) investigated how adults processed two-digit integer numbers using both a simultaneous and a sequential 2AFC task. They observed a congruency effect in the simultaneous task, suggesting that participants were processing information in a fragmented way. In contrast, no congruency effect was observed in the sequential task, suggesting that participants processed information holistically. This finding suggests that the format of 2AFC tasks influences how people integrate stimuli components into holistic information. Nevertheless, other studies have found that task format has no influence on information processing. For instance, Moeller and colleagues (2013), also investigating two-digit integer number processing, found a congruency effect in both simultaneous and sequential 2AFC tasks, suggesting that fragmented information processing does not change as a function of 2AFC task format. Considering these studies together, there is no clear guidance on when to use each version of the 2AFC task, and we still do not fully understand how the task format influences information processing. In particular, since these studies were limited to integer numbers, we do not know how task format influences other types of stimuli.

## Current study

The current study investigates how the format of 2AFC tasks influences information processing, using multicomponent numbers that may serve as a model for other comparisons of sequential versus simultaneous 2AFC paradigms. Specifically, we examined whether the distance effect and the congruency effect in two-digit integer, fraction, and decimal comparisons are consistent across 2AFC task formats or vary depending on whether the task is simultaneous or sequential.

Using a within-subjects design, we presented young adults with both simultaneous and sequential versions of two-digit integer, fraction, and decimal 2AFC comparison tasks. In these tasks, comparison pairs varied in numerical distance (ranging from small to large absolute differences) and congruency (i.e., when holistic and component-based magnitudes are congruent vs. incongruent). We preregistered three main hypotheses. First, we expected the distance effect to be driven by absolute numerical differences (i.e., greater number – smaller number) across two-digit integers, fractions, and decimals in both simultaneous and sequential tasks if people consistently processed information holistically, regardless of 2AFC format. In contrast, if people consistently processed stimuli in a fragmented way, regardless of 2AFC format, we expected a strong congruency effect in both simultaneous and sequential tasks, with individuals relying on specific numerical components (e.g., ones vs. tens for place value of integers, numerator vs. denominator for fractions, and tenths vs. hundredths for decimals). Finally, we expected differences in the strength of the distance and congruency effects between simultaneous and sequential tasks if the way people engage in holistic versus fragmented processing is influenced by 2AFC task format.

## Methods

### Participants

Participants were students enrolled in a psychology course recruited through the university’s research participant pool online platform and through flyers distributed throughout the university's psychology building. Participants were compensated with research credits. The inclusion criteria required participants to be healthy native English speakers, aged 18 to 35 years, with normal or corrected-to-normal vision and no self-reported color blindness.

We preregistered a sample size of 150 participants, along with exclusion criteria requiring (1) at least 50% trials with response time (RT) falling within three standard deviations from participants’ mean RT and (2) accuracy above 50% to ensure data quality. Anticipating some exclusions, we oversampled to achieve the target sample size. A total of 181 students enrolled in the study, but 15 did not finish the experiment due to technical failures (i.e., computer crashes, *n* = 7), experimenter error (*n* = 5), or participant withdrawal (*n* = 3). Thus, a total of 166 participants completed both simultaneous and sequential tasks. We then applied the preregistered exclusion criteria for each task format and retained only participants with high data quality in both simultaneous and sequential tasks. Since two-digit integer, fraction, and decimal comparisons may vary in difficulty, we applied exclusions separately for each number type, resulting in slight variations in final sample sizes: 162 participants for the two-digit integer task, 160 for the fraction task, and 156 for the decimal task.

Participants’ demographic characteristics are reported in Table 1, based on the largest sample (*n* = 162).

**Table 1:** Participants’ demographic characteristics (*n* = 162)

Measure	Mean (range)	***SD***
**Age**	19.17 (18–26)	1.24
	**No. male (%)**	**No. female (%)**
**Sex**	48 (30)	114 (70)

### General procedures

Participants completed simultaneous and sequential 2AFC tasks measuring multicomponent number processing with two-digit integers, fractions, and decimals. In each task, participants indicated which of two numbers had a *greater magnitude*. The entire session lasted 2 hours and was conducted in our lab by trained research assistants following standardized scripts (available on OSF, https://osf.io/yzgws/overview?view_only=ca841e5901a74320a3e2145ee5b1d37e). First, participants provided oral consent to participate and received instructions. Then they completed the experiments, with short breaks offered between tasks, followed by a sociodemographic questionnaire.

### Stimuli and design

**Fig. 1:**
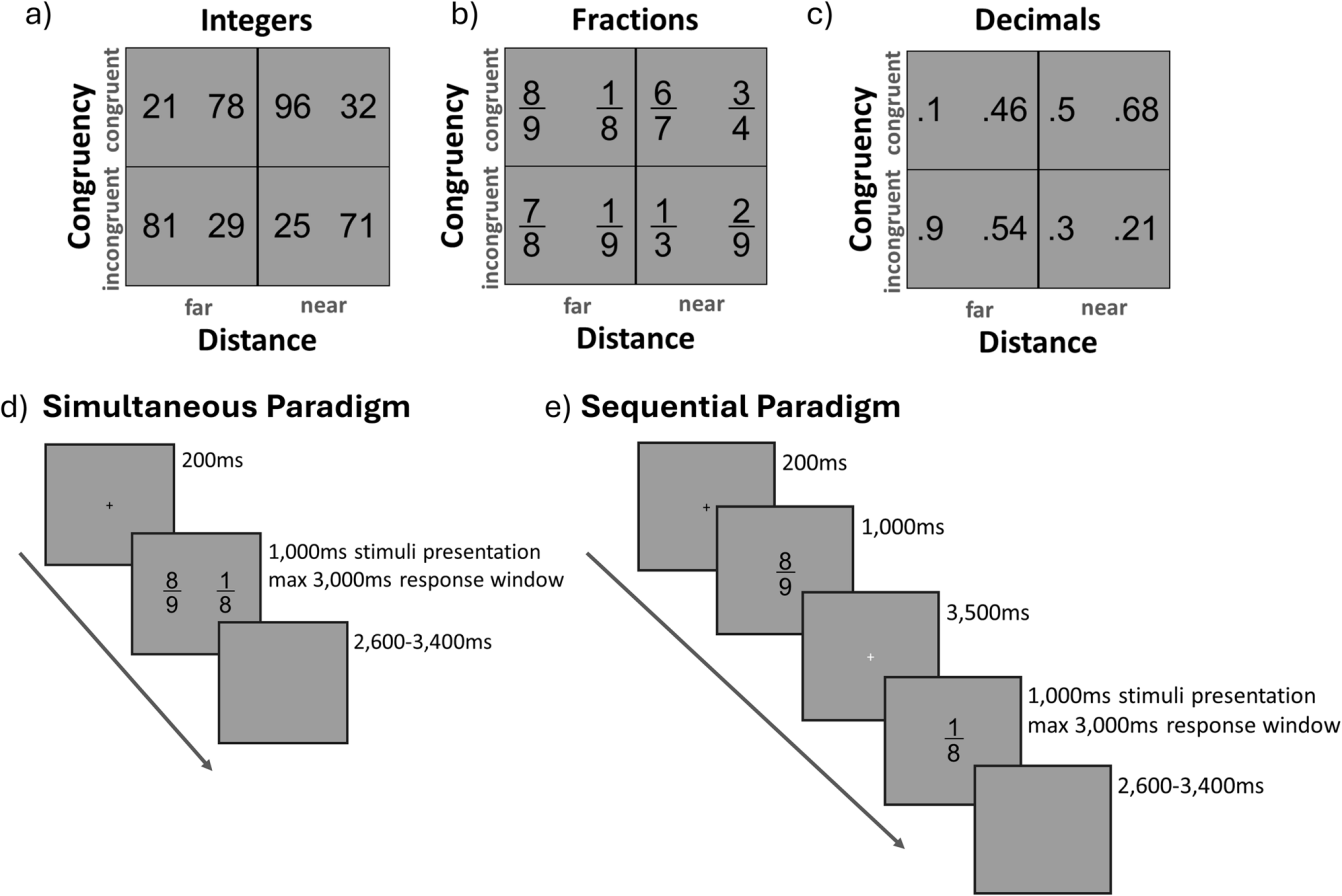
Stimuli used in each task and example of simultaneous and sequential paradigms. *Note.* This figure illustrates how stimuli varied based on distance (near vs. far) and congruency (congruent vs. incongruent) in **a** two-digit integer comparisons, **b** fraction comparisons, and **c** decimal comparisons. Examples of simultaneous and sequential paradigms are displayed on panels **d** and **e**. In simultaneous paradigms (**d**), both stimuli were shown on the same screen; in sequential paradigms (**e**), stimuli were shown on separate screens

In both the simultaneous and sequential tasks, participants were presented with a total of 40 unique pairs of each number type. These pairs varied in distance and congruency (see Fig. 1a–c). In each task, 20 pairs were separated by a near distance, with 10 congruent trials and 10 incongruent trials. The remaining 20 pairs were separated by a far distance, also with 10 congruent trials and 10 incongruent trials. The pairs were shown twice with a balanced order of presentation (i.e., whether the greater number was shown to the left/right in the simultaneous task, or as first/second in the sequential task), resulting in a total of 80 trials for each number type.

The experiments were programmed in PsychoPy, counterbalanced across participants for both number type order (two-digit integers, fractions, and decimals) and task format order (simultaneous, sequential), and presented on a 27-inch Apple iMac. Two-digit integers, fractions, and decimals were displayed in black Arial font (height = 0.3 normalized units, RGB = 0, 0, 0) against a gray background (RGB = 157, 157, 157). Participants indicated their responses using a standard US QWERTY keyboard.

In the simultaneous tasks (see Fig. 1d), stimuli were shown on the left (*X* = −0.35, *Y* = 0) and right sides of the screen (*X* = 0.35, *Y* = 0). Each task began with three practice trials that included feedback, followed by testing trials without feedback. At the start of each trial, participants saw a fixation cross for 200 ms. Then, they were presented with two numbers on the screen for 1,000 ms. They were instructed to press “z” if the number on the left side of the screen was greater and “m” if the number on the right was greater. Participants had up to 3,000 ms to respond. Trials were separated by a mean intertrial interval of 3,000 ms (range 2,600–3,400 ms).

In the sequential tasks (see Fig. 1e), stimuli were presented at the center (*X* = 0, *Y* = 0) of consecutive screens. Each task began with three practice trials that included feedback, followed by testing trials without feedback. At the start of each trial, participants saw a fixation cross for 200 ms. Then the first number was presented (1,000 ms), followed by an inter-stimulus interval (3,500 ms), and then the second number (1,000 ms). We used a 3,500-ms inter-stimulus interval to give participants sufficient time to process the first stimulus before seeing the second stimulus, in line with previous implementations of this paradigm (e.g., DeWolf et al., 2016). Participants were instructed to respond as soon as they saw the second number, by pressing “z” if the first number was greater and “m” if the second number was greater. Participants had up to 3,000 ms to respond. Trials were separated by a mean intertrial interval of 3,000 ms (range 2,600–3,400 ms).

Non-responses were classified as errors. We iteratively trimmed participants' response times (RT) for each task at the individual level. We calculated the mean RT for each participant and excluded any RTs that were slower or faster than three standard deviations from the individual means. Additionally, we excluded trials with RTs faster than 200 ms.

#### **Integer comparison**

In the integer number comparison task, participants were presented with a pair of two-digit integer numbers and instructed to identify which was greater. We developed 40 pairs of two-digit integers based on stimuli created by Nuerk and colleagues (2004). All pairs consisted of numbers with non-shared units and decades (e.g., 24 vs. 36). Holistic distance (i.e., larger number − smaller number) ranged from 5 to 67 (*near distances*: distances < 26, mean = 15; *far distance*s: distances > 26, mean = 55). Additionally, pairs could be congruent or incongruent. Congruent pairs had units whose magnitudes were compatible with the holistic magnitude of the stimulus, such that the greater number also had a greater unit (e.g., 87 vs. 24, where 87 > 24 and 7 > 4). Incongruent pairs had units whose magnitudes were incompatible with the holistic magnitude of the stimulus, such that the greater number had a smaller unit (e.g., 84 vs. 27, where 84 > 27 but 4 < 7). Average split-half reliability was high in both simultaneous (*r* = .94) and sequential (*r* = .96) tasks. The full list of stimuli is shown in Supplementary Information (S1).

#### **Fraction comparison**

In the fraction comparison task, participants were presented with two fractions and instructed to identify which one was greater. We adapted 40 fraction pairs from comparison lists developed by Binzak and Hubbard (2020) and Dewolf and colleagues (2016). These pairs consisted of proper fractions composed of single-digit numerators and denominators. The fractions were separated by either a near or a far holistic distance, which ranged from 0.10 to 0.76 (*near distances*: distances < 0.33, mean = 0.15; *far distances*: distances > 0.33, mean = 0.56). Pairs could be congruent or incongruent. In congruent pairs, (1) the greater fraction also had a greater numerator and denominator (e.g., 7/8 vs. 3/4, 7 > 3 and 8 > 4), or (2) both fractions shared the same denominator and the greater fraction had a great numerator (e.g., 8/9 vs. 4/9, 8 > 4). Incongruent trials were those where this pattern did not apply, such that (1) the greater fraction had a smaller numerator and denominator (e.g., 3/8 vs. 1/2, 3 > 1 and 8 > 2), or (2) the greater fraction had a smaller denominator (e.g., 2/9 vs. 2/3, 9 > 3; 1/8 vs. 6/7, 8 > 7). Average split-half reliability was high in both simultaneous (*r* = .91) and sequential (*r* = .95) tasks. The full list of stimuli is shown in Supplementary Information (S2).

#### **Decimal comparison**

In the decimal comparison task, participants were presented with two decimal numbers and instructed to identify which one was greater. We adapted 40 pairs of decimals from a comparison list created by Rosenberg-Lee and colleagues (2023). In one number of each pair, only one digit was displayed after the decimal point. In the other decimal number, either two digits (20 pairs; extracted from Robsenberg-Lee et al., 2023) or three digits (20 pairs; adapted items) were displayed after the decimal point. The decimals with three digits after the decimal point represented the same magnitude as those with two digits; however, they included a zero as the third digit (e.g., two digits after the point = 0.31; three digits after the point = 0.310). The decimals were separated by either a near distance or a far distance, which ranged from 0.09 to 0.90 (*near distance*s: all near distances = 0.09; *far distances*: distances > 0.9, mean = 0.27). Pairs could be congruent, where the decimal with more digits after the decimal point represented a larger magnitude (e.g., 0.5 vs. 0.680), or incongruent, where the decimal with fewer digits after the decimal point represented a larger magnitude (e.g., 0.5 vs. 0.410). Average split-half reliability was acceptable in both simultaneous (*r* = .93) and sequential (*r* = .94) tasks. The full list of stimuli is shown in Supplementary Information (S3).

### Analyses

We preregistered three main analyses to investigate how task format influences performance in two-digit integer, fraction, and decimal comparisons: (1) comparisons of distance and congruency effects between simultaneous and sequential tasks, (2) correlations between distance and congruency effects in simultaneous and sequential tasks, and (3) linear mixed models examining how task format, distance, congruency, and the interaction between these terms, influence performance. Given that the two-digit integer, fraction, and decimal tasks assess distinct numerical representations, we conducted analyses separately for each number type rather than combining them into a single model.

In the comparison and correlation analyses, we used calculated indices of distance and congruency effects. These indices are calculated relative to each participant’s overall task performance, which makes them less sensitive to fluctuations in average RT or accuracy across different task formats. We used these indices to facilitate comparison with the broader literature on numerical cognition and 2AFC tasks, as they are widely used to quantify performance in these paradigms (e.g., Bausenhart et al., 2021; Felisatti et al., 2024; Goffin & Ansari, 2016; Shichel & Goldfarb, 2022). For the distance effect, we computed a standardized score representing the cost of comparing near-distance pairs relative to far-distance pairs:

$$\begin{array}{c}Accuracy\;distance\;effect=\left(\frac{{accuracy}_{far}-{accuracy}_{near}}{mean\;accuracy}\right)\\RTdistance\;effect\hspace{0.17em}=\hspace{0.17em}\left(\frac{{RT}_{near}-{RT}_{far}}{mean\;RT}\right)\end{array}$$

Here, a positive value indicates greater accuracy and faster responses for far distances than near distances (i.e., typical distance effect), while a negative value suggests the opposite (i.e., reversed distance effect). Values closer to zero indicate similar accuracy and RT for near and far distances.

To quantify the congruency effect, we used a similar standardized score, but calculated with congruent and incongruent trials instead of near and far distances:

$$\begin{array}{c}Accuracy\;congruency\;effect=\left(\frac{{accuracy}_{congruent}-{accuracy}_{incongruent}}{mean\;accuracy}\right)\\RT\;congruency\;effect=\left(\frac{{RT}_{incongruent}-{RT}_{congruent}}{mean\;RT}\right)\end{array}$$

A positive value indicates greater accuracy and faster responses for congruent compared to incongruent items (i.e., a typical congruency effect). A negative value indicates greater accuracy and faster responses for incongruent compared to congruent items (i.e., reversed congruency effect). Values closer to zero suggest similar accuracy and RT for congruent and incongruent items.

We contrasted the distance and congruency effects (calculated as described above) between simultaneous and sequential tasks using paired *t*-tests. We then examined bivariate correlations between distance and congruency effects across task formats. We applied Benjamini–Hochberg correction to control for multiple tests (four tests, comprising distance and congruency effects calculated with accuracy and RT within each number type).

Since these comparisons and correlations rely on differences between distance and congruency effects, this approach may not capture individual differences. Thus, we also preregistered linear mixed-effects models. We ran two separate models for each of the three magnitude types: one with item-level accuracy as the outcome, and one with item-level RT as the outcome. Task format (dummy coded: simultaneous = 1, sequential = 0), holistic distance (scaled), congruency (dummy coded: incongruent = 1, congruent = 0), and their interactions were included as fixed effects. To account for inter-individual variability, we specified a random intercept for each participant and a random slope for the interaction between holistic distance and congruency, allowing this effect to vary across participants.

For the accuracy analyses, we employed generalized linear mixed models with a binomial error distribution and a logistic link function. To improve the accuracy model convergence, we used the *bobyqa* (bound optimization BY quadratic approximation) optimizer, with a maximum of 100,000 function evaluations. *Bobyqa* is a derivative-free optimization algorithm that approximates the objective function using a quadratic model, making it well suited for complex mixed-effects models. For the RT analyses, we employed linear mixed-effects models with a Gaussian error distribution and an identity link function. We implemented these analyses in R using the *glmer* and *lmer* functions from the lme4 package (Bates, 2018), and estimated *p*-values using the lmerTest package (Kuznetsova et al., 2022). When significant interactions were observed, we followed up with post hoc simple slopes analyses.

## Results

### Descriptive statistics

Table 2 presents participants’ mean accuracy and response time (RT) across all trials, distance levels, and congruency conditions in simultaneous and sequential two-digit integer, fraction, and decimal comparisons. It also shows the size effects of the contrast between near versus far and congruent versus incongruent conditions within each number type and task format. Overall, participants were more accurate and faster when comparing far distances relative to near distances and congruent trials relative to incongruent trials. However, in the sequential decimal task, participants showed slower RT for congruent trials than incongruent trials, demonstrating a reversed congruency effect.

**Table 2:** Descriptive statistics

Experimental condition	Simultaneous		Sequential	
	*M*	*SD*	*M*	*SD*
**Integer comparison (*n* = 162)**				
***Accuracy (0–1)***				
All items	0.97	0.16	0.97	0.17
Far distance	0.99	0.09	0.98	0.14
Near distance	0.96	0.21	0.96	0.20
Distance effect	0.04	0.04	0.03	0.05
*Near vs. far within task format comparison*	*t*(*df* = 161) =10.96, *p* < .001*, Cohen’s *d* = 1.01		*t*(*df* = 161) = 6.89, *p* < .001*, Cohen’s *d* = 0.63	
Congruent pairs	0.99	0.09	0.97	0.16
Incongruent pairs	0.96	0.21	0.97	0.18
Congruency effect	0.04	0.03	0.01	0.04
*Congruent vs. incongruent within task format comparison*	*t*(*df* = 161) = 11.37, *p* < .001*, Cohen’s *d* = 1.00		*t*(*df* = 161) = 1.98, *p* = .008*, Cohen’s *d* = 0.17	
***RT (ms)***				
All items	664.01	226.25	676.10	364.83
Far distance	613.87	203.60	627.73	352.17
Near distance	716.66	236.67	726.21	370.93
Distance effect	0.15	0.07	0.15	0.10
*Near vs. far within task format comparison*	*t*(*df* = 161) = 23.25, *p* < .001*, Cohen’s *d* = 0.62		*t*(*df* = 161) = 18.77, *p* < .001*, Cohen’s *d* = 0.33	
Congruent pairs	641.14	216.13	669.50	367.50
Incongruent pairs	687.93	234.02	682.74	362.04
Congruency effect	0.07	0.05	0.02	0.07
*Congruent vs. incongruent within task format comparison*	*t*(*df* = 161) = 14.64, *p* < .001*, Cohen’s *d* = 0.30		*t*(*df* = 161) = 3.36, *p* < .001*, Cohen’s *d* = 0.05	
**Fraction comparison (*n* = 160)**				
***Accuracy (0–1)***				
All items	0.94	0.23	0.93	0.25
Far distance	0.98	0.14	0.97	0.17
Near distance	0.90	0.30	0.90	0.30
Distance effect	0.08	0.06	0.08	0.08
*Near vs. far within task format comparison*	*t*(*df* = 159) = 18.11, *p* < .001*, Cohen’s *d* = 1.37		*t*(*df* = 159) = 13.85, *p* < .001*, Cohen’s *d* = 1.08	
Congruent pairs	0.96	0.19	0.94	0.23
Incongruent pairs	0.92	0.27	0.93	0.26
Congruency effect	0.05	0.08	0.02	0.07
*Congruent vs. incongruent within task format comparison*	*t*(*df* = 159) = 8.05, *p* < .001*, Cohen’s *d* = 0.80		*t*(*df* = 159) = 3.15, *p* = .008*, Cohen’s *d* = 0.27	
***RT (ms)***				
All items	1068.69	419.02	805.19	445.41
Far distance	968.89	364.70	728.96	401.34
Near distance	1180.22	446.79	888.59	475.31
Distance effect	0.19	0.09	0.19	0.10
*Near vs. far within task format comparison*	*t*(*df* = 159) = 22.08, *p* < .001*, Cohen’s *d* = 0.77		*t*(*df* = 159) = 18.22, *p* < .001*, Cohen’s *d* = 0.43	
Congruent pairs	1014.11	408.26	762.59	422.35
Incongruent pairs	1126.49	422.53	848.74	463.81
Congruency effect	0.10	0.09	0.10	0.10
*Congruent vs. incongruent within task format comparison*	*t*(*df* = 159) = 13.45, *p* < .001*, Cohen’s *d* = 0.43		*t*(*df* = 159) = 11.01, *p* < .001*, Cohen’s *d* = 0.25	
**Decimal comparison (*n* = 156)**				
***Accuracy (0–1)***				
All items	0.97	0.16	0.95	0.22
Far distance	0.97	0.18	0.96	0.20
Near distance	0.98	0.15	0.94	0.23
Distance effect	−0.01	0.04	0.01	0.05
*Near vs. far within task format comparison*	*t*(*df* = 155) = −3.18, *p* = .007*, Cohen’s *d* = −0.27		*t*(*df* = 155) = 3.74, *p* < .001*, Cohen’s *d* = 0.27	
Congruent pairs	0.99	0.10	0.95	0.23
Incongruent pairs	0.96	0.20	0.95	0.22
Congruency effect	0.03	0.05	0.00	0.06
*Congruent vs. incongruent within task format comparison*	*t*(*df* = 155) = 8.51, *p* < .001*, Cohen’s *d* = 0.79		*t*(*df* = 155) = 0.68, *p* = 1*, Cohen’s *d* = 0.05	
***RT (ms)***				
All items	805.51	233.44	731.03	303.00
Far distance	803.96	236.09	712.29	301.55
Near distance	807.05	230.80	750.14	303.31
Distance effect	0.01	0.05	0.05	0.08
*Near vs. far within task format comparison*	*t*(*df* = 155) = 1.03, *p* = 1*, Cohen’s *d = *0.02		*t*(*df* = 155) = 7.08, *p* < .001*, Cohen’s *d* = 0.18	
Congruent pairs	804.90	241.45	748.17	306.47
Incongruent pairs	806.14	224.93	713.98	298.56
Congruency effect	0.00	0.07	−0.05	0.07
*Congruent vs. incongruent within task format comparison*	*t*(*df* = 155) = −0.14 *p* = 1*, Cohen’s *d* = 0.00		*t*(*df* = 155) = −8.00, *p* < .001*, Cohen’s *d* = −0.17	

*p-values corrected for four multiple comparisons using the Benjamini–Hochberg method. Uncorrected values are shown on OSF

### How performance in the simultaneous tasks differs from performance in the sequential tasks

First, we conducted preregistered comparisons between the distance effect and the congruency effect in simultaneous and sequential tasks. These analyses indicate whether the distance and congruency effects are consistent or vary between the two task formats. Results are summarized in Table 3 and described below. Overall, the results revealed differences between the simultaneous and sequential tasks, particularly in the congruency effect, indicating that task format influences multicomponent number processing.

**Table 3:** Summary of findings from comparison analyses

Effect	Integers	Fractions	Decimals
**Distance**	Simultaneous = Sequential	Simultaneous = Sequential	Simultaneous < Sequential
**Congruency**	Simultaneous > Sequential	Simultaneous > Sequential^a^	Simultaneous > Sequential^a^

= indicates no significant differences across task formats. > indicates stronger effect in simultaneous than sequential task. < indicates stronger effect in sequential than simultaneous task. ^a^ Effect only observed with accuracy rates

### Distance effect

#### Integers

**Fig. 2:**
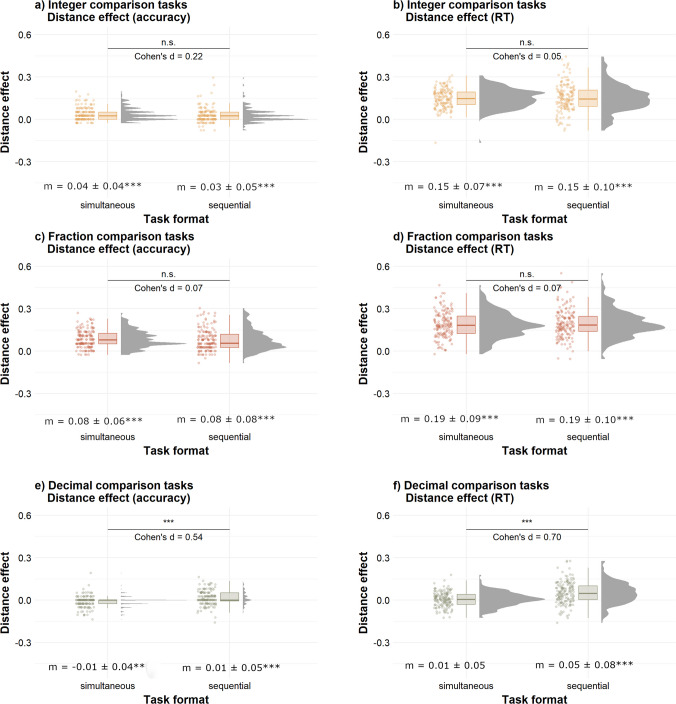
Distance effects in sequential and simultaneous comparison tasks. *Note.* This figure illustrates the distribution of distance effects calculated with accuracy (left panels) and RT (right panels) in the two-digit integer comparison tasks (panels **a** and **b**, top), fraction comparison tasks (panels **c** and **d**, middle), and decimal comparison tasks (panels **e** and **f**, bottom). For accuracy, the distance effect was calculated as *distance effect = (accuracy in far-distance trials − accuracy in near-distance trials)/mean accuracy.* For RT, the distance effect was calculated as *distance effect = (RT in near-distance trials − RT in far-distance trials)/mean RT*. Cohen’s *d* values shown in the middle indicate between-format comparisons (n.s. = nonsignificant; **p < .001). The mean congruency effect (± *SD*) is shown at the bottom of each figure, with results of *t*-tests against zero represented by asterisks (**p < .01, ***p < .001) and comprehensive results shown on OSF

As illustrated in Fig. 2a and b, distance effects measured with both accuracy, *t*(161) = 2.06, *p* = .166, *d* = 0.22, and RT, *t*(161) = 0.41, *p* = 1.00, *d = *0.05, did not differ across simultaneous and sequential tasks when controlling for multiple comparisons. These results suggest that the distance effect was similarly elicited in simultaneous and sequential two-digit integer comparisons.

#### **Fractions**

As illustrated in Fig. 2c and d, distance effects measured with both accuracy, *t*(159) = 0.76, *p* = .446, *d* = 0.07, and RT, *t*(159) = 0.61, *p* = .544, *d* = 0.07, did not differ across simultaneous and sequential tasks. This result suggests that the distance effect was similarly elicited in simultaneous and sequential fraction comparisons.

#### **Decimals**

As illustrated in Fig. 2e and f, participants showed stronger distance effects in the sequential than in the simultaneous task, when both accuracy, *t*(155) = 4.66, *p* < .001, *d* = 0.54, and RT, *t*(155) = 6.57, *p* < .001, *d* = 0.70, were considered. These results remained significant when correcting for multiple comparisons (all *p*s < .001), and suggest that task format influenced the strength of the distance effect in decimal comparisons.

### Congruency effect

#### **Integers**

**Fig. 3:**
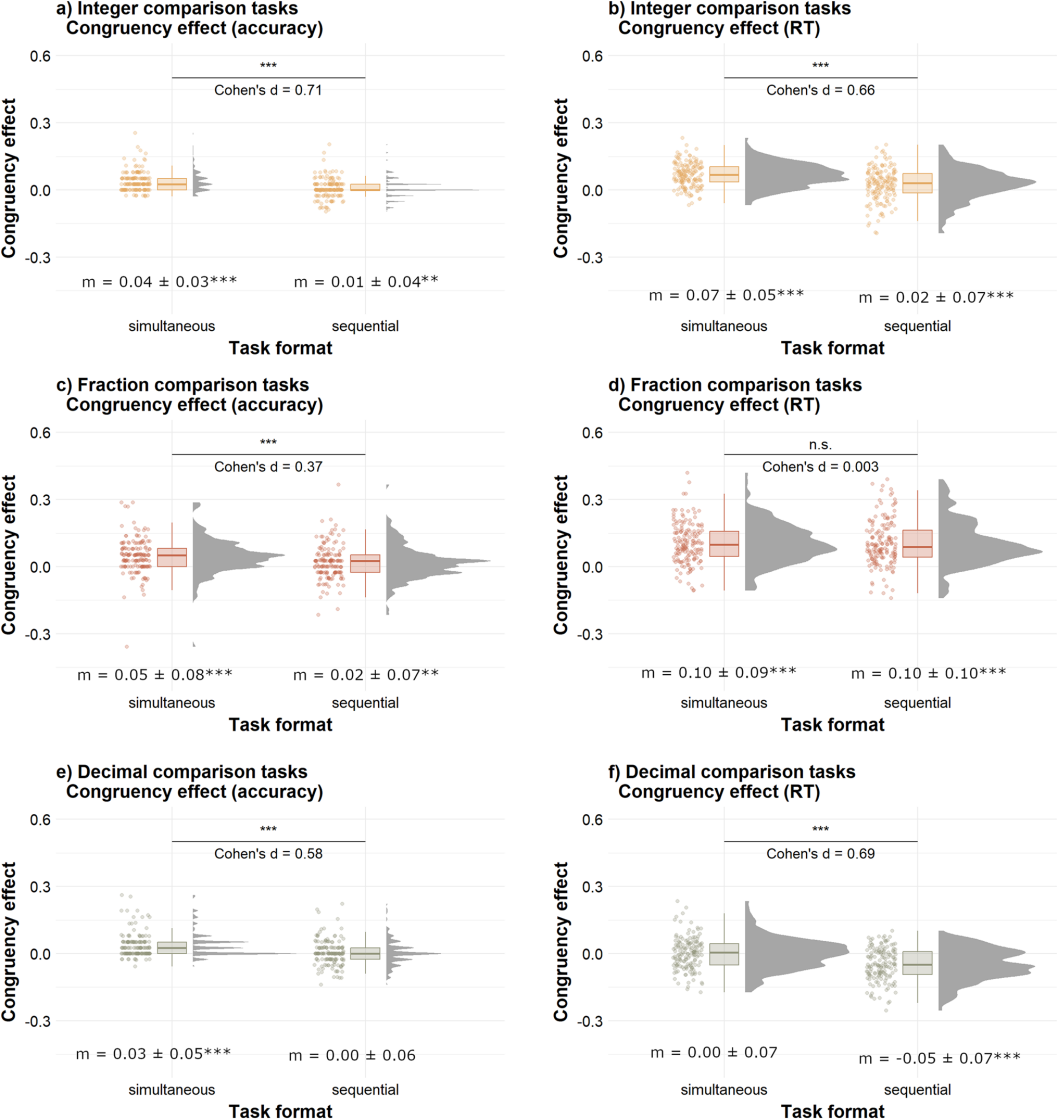
Congruency effects in sequential and simultaneous comparison tasks. *Note.* This figure illustrates the distribution of congruency effects calculated with accuracy (left panels) and RT (right panels) in the two-digit integer comparison tasks (panels **a** and **b**), fraction comparison tasks (panels **c** and **d**), and decimal comparison tasks (panels **e** and **f**). For accuracy, the congruency effect was calculated as *congruency effect = (accuracy in congruent trials − accuracy in incongruent trials)/mean accuracy.* For RT, distance effect was calculated as *congruency effect = (RT in incongruent trials − RT in congruent trials)/mean RT*. Cohen’s *d* values shown in the middle indicate between-format comparisons (n.s. = nonsignificant; **p < .001). The mean congruency effect (± *SD*) is shown at the bottom of each figure, with results of *t*-tests against zero represented by asterisks (**p < .01, ***p < .001) and comprehensive results shown on OSF

As illustrated in Fig. 3a and b, the congruency effect measured with both accuracy, *t*(161) = 5.91, *p* < .001, *d* = 0.71, and RT, *t*(161) = 6.21, *p < *.001, *d* = 0.66, was stronger in the simultaneous task than in the sequential task. These results remained significant when correcting for multiple comparisons (*p*s < .001), and suggest that task format influenced the strength of the congruency effect in integer comparisons.

#### **Fractions**

As illustrated in Fig. 3c and d, the congruency effect measured with accuracy was stronger in the simultaneous task than in the sequential task, *t*(159) = 4.42, *p* < .001, *d* = 0.37. To explore whether this result was influenced by two outliers, we reanalyzed the data without them, and the effect remained significant, *t*(157) = 4.94, *p* < .001, *d* = 0.45. The results also remained significant when correcting for multiple comparisons (*p*s < .001), and suggest that task format influenced the strength of the congruency effect in fraction comparisons. However, the congruency effect measured with RT did not differ across task formats, *t*(159) = 0.03, *p* = .979, *d* = 0.003.

#### Decimals

As illustrated in Fig. 3e and f, the congruency effect measured with accuracy was stronger in the simultaneous task than in the sequential task, *t*(155) = 5.14, *p* < .001, *d* = 0.58. These results also remained significant when correcting for multiple comparisons (*p*s < .001). When the congruency effect was measured with RT, there was also a difference across simultaneous and sequential tasks, *t*(155) = 7.05, *p < *.001, *d* = 0.69, which remained significant when correcting for multiple comparisons (*p*s < .001). However, this difference was driven by a reversed congruency effect in the sequential task contrasted with a congruency effect close to zero in the simultaneous task. These results suggest that task format influenced the strength and direction of the congruency effect in decimal comparisons.

### How performance in the simultaneous tasks correlates with performance in the sequential tasks

The comparison analyses reported above suggested differences in distance and congruency effects across simultaneous and sequential task formats. However, even when we observe differences in mean performance across these tasks, they might still capture individual differences in a similar way. For example, a participant with the lowest accuracy in the simultaneous task relative to other participants may also show the lowest accuracy in the sequential task.

To explore how simultaneous and sequential tasks capture individual differences, we conducted bivariate correlations, as preregistered. If simultaneous and sequential tasks captured individual differences similarly, we would expect moderate to strong positive correlations. In contrast, a negative correlation would suggest that participants with stronger effects in one task format exhibit weaker or reversed effects in the other. Finally, weak or nonsignificant correlations would suggest substantial variation in participants’ distance and congruency effects across task formats.

We summarize the results in Table 4 and describe them in detail below. Full correlation matrices can be found on the project OSF page. Overall, the results revealed that the simultaneous and sequential tasks captured participants’ variance differently, corroborating the hypothesis that task format influences multicomponent number processing. Except for fraction comparisons, the distance effects elicited by the simultaneous and sequential tasks were not significantly correlated. Similarly, the congruency effects were not correlated for two-digit integer comparisons and were weakly correlated for decimal comparisons; however, they were moderately correlated for fraction comparisons.

**Table 4:** Summary of findings from correlation analyses

Effect	Integers	Fractions	Decimals
**Distance**	−	↑^*^	−
**Congruency**	−	↑↑^*^	↑^+^

− indicates no significant correlation; ↑ indicates weak, positive correlation; ↑↑ indicates moderate, positive correlation. *Effect only observed with accuracy rates; ^+^Effect only observed with RT.

### Distance effect

#### **Integers**

**Fig. 4:**
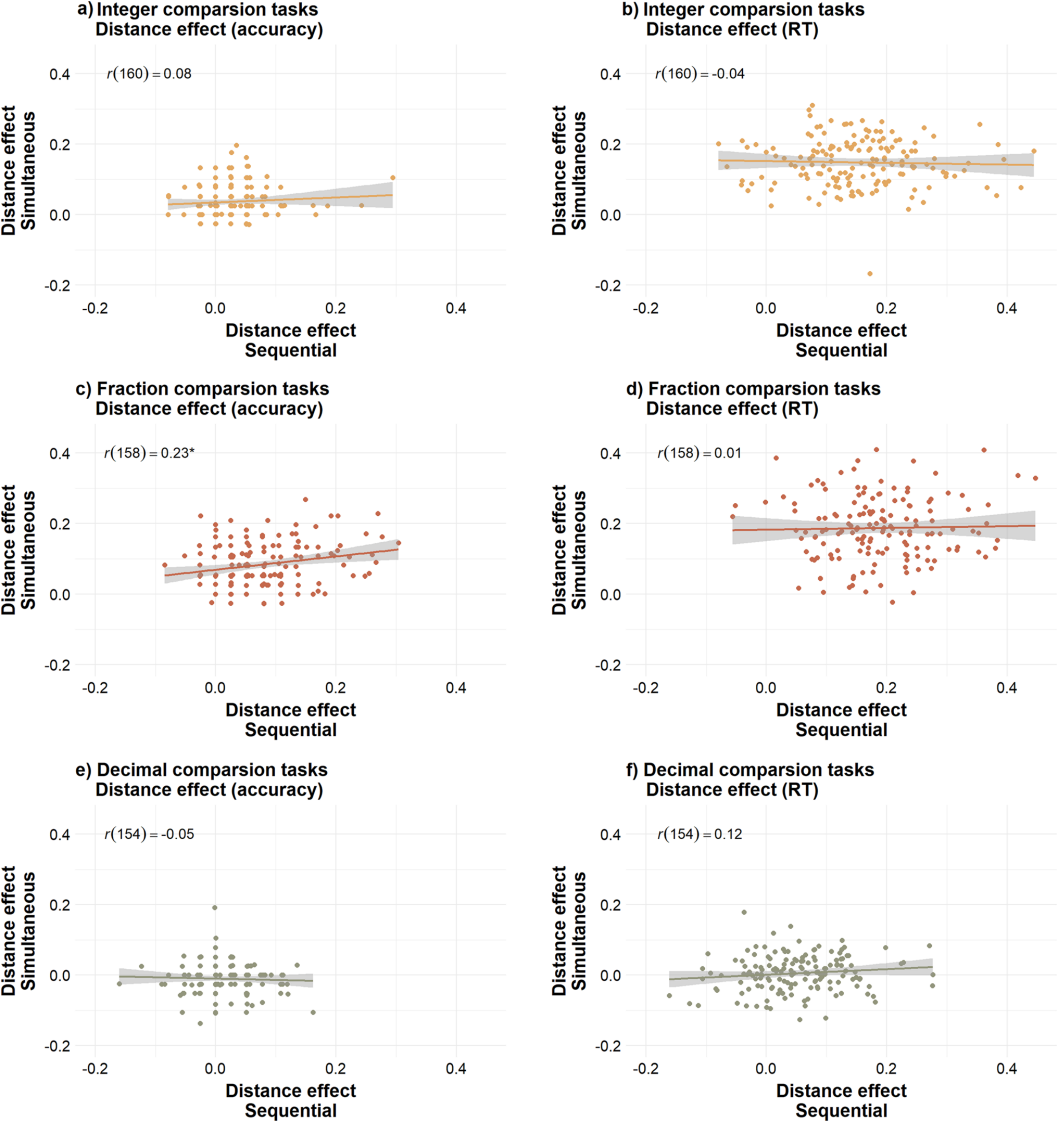
Correlations between distance effects in simultaneous and sequential comparison tasks. This figure shows the correlations between distance effects measured in the simultaneous and sequential versions of the two-digit integer comparison tasks (panels **a** and **b**), fraction comparison tasks (panels **c** and **d**), and decimal comparison tasks (panels **e** and **f**). The panels on the left show the distance effects calculated using accuracy, as *distance effect = (accuracy in far-distance trials − accuracy in near-distance trials)/mean accuracy*. The panels on the right show the distance effects calculated using RT, as *distance effect = (RT in near-distance trials − RT in far-distance trials)/mean RT*.

As illustrated in Fig. 4a and b, distance effects in simultaneous and sequential tasks were not significantly correlated when measured with either accuracy, *r*(160) = 0.08, *p* = .310, or RT, *r*(160) = −0.04, *p* = .650. These results indicate that simultaneous and sequential tasks may not equally capture individual differences in two-digit integer distance effects.

#### **Fractions**

As illustrated in Fig. 4c and d, distance effects indexed in simultaneous and sequential tasks showed positive but weak correlation when accuracy was considered, *r*(158) = 0.23, *p* = .003. This correlation remained significant after applying Benjamini–Hochberg correction (*p* = .012). Distance effects were not correlated when RT was considered, *r*(158) = 0.01, *p* = 0.895. These results indicate that simultaneous and sequential tasks may not equally capture individual differences in fraction distance effects.

#### **Decimals**

As illustrated in Fig. 4 e and f, the distance effects in sequential and simultaneous tasks were not significantly correlated when measured with either accuracy, *r*(154) = −0.05, *p* = .559, or RT, *r*(154) = 0.12, *p* = .129. These results indicate that simultaneous and sequential tasks may not equally capture individual differences in decimal distance effects.

### Congruency effect

#### **Integers**

**Fig. 5:**
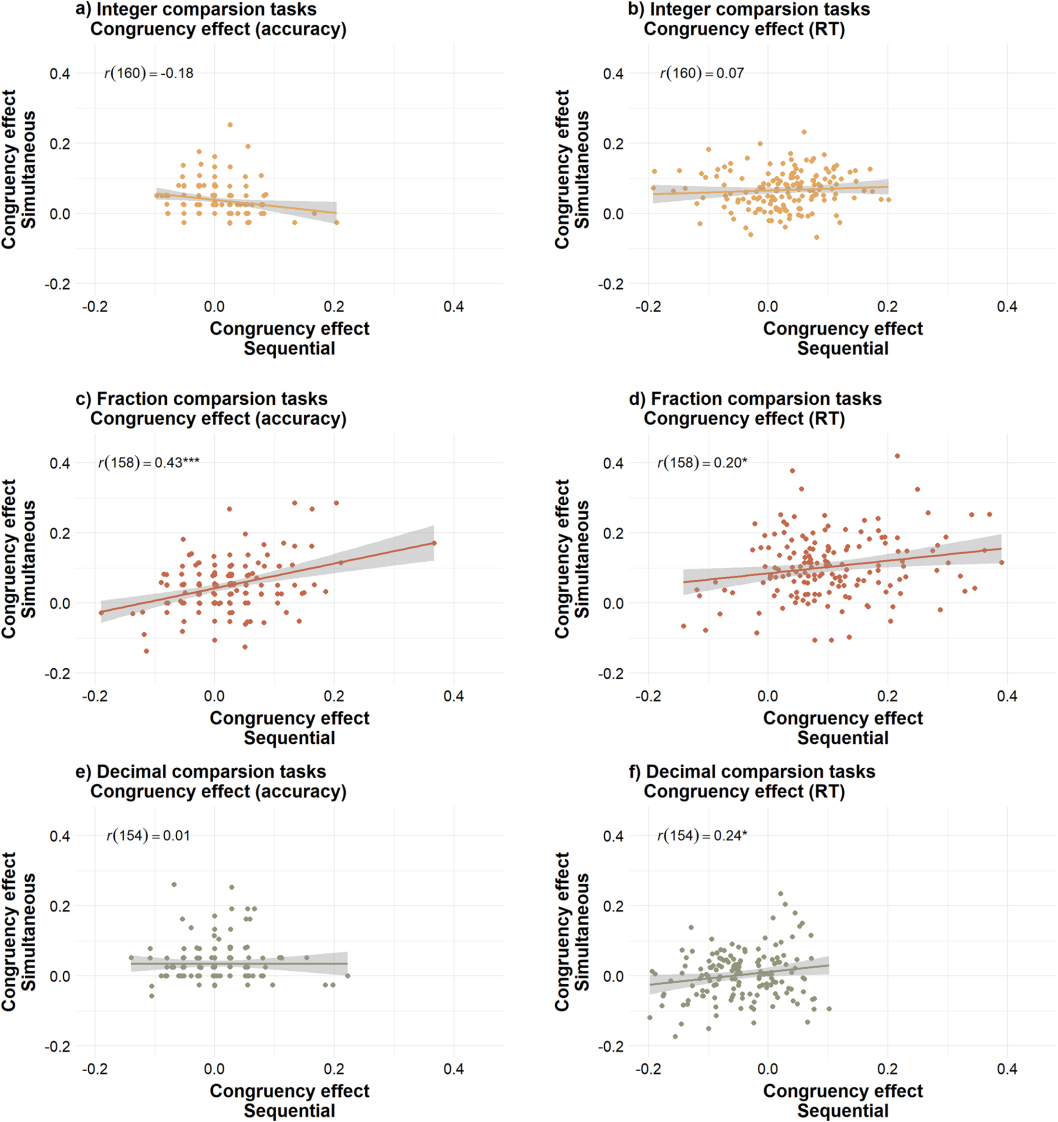
Correlations between congruency effects in simultaneous and sequential comparison tasks. *Note.* This figure shows the correlations between congruency effects measured in the simultaneous and sequential versions of the two-digit integer comparison tasks (panels **a** and **b**), fraction comparison tasks (panels **c** and **d**), and decimal comparison tasks (panels **e** and **f**). The panels on the left show the congruency effects calculated using accuracy, as *congruency effect = (accuracy in congruent trials − accuracy in incongruent trials)/mean accuracy*. The panels on the right show the congruency effects calculated using RT, as *congruency effect = (RT in incongruent trials − RT in congruent trials)/mean RT*

As illustrated in Fig. 5a and b, there was a weak negative correlation between congruency effects measured with accuracy in simultaneous and sequential tasks, *r*(160) = −0.18, *p = *.021. However, this correlation did not remain significant when applying Benjamini–Hochberg correction (*p* = .083). There was no correlation between congruency effects measured with RT in simultaneous and sequential tasks, *r*(160) = 0.07, *p* = .363 . These results indicate that simultaneous and sequential tasks may not equally capture individual differences in two-digit integer congruency effects.

#### **Fractions**

As illustrated in Fig. 5c and d, there was a positive correlation between congruency effects in simultaneous and sequential tasks, measured with both accuracy, *r*(158) = 0.43, *p < *.001, and RT, *r*(158) = 0.20, *p* = .010. These correlations remained significant after applying Benjamini–Hochberg correction (accuracy *p* < .001, RT *p* = .042). These results indicate that simultaneous and sequential tasks may similarly capture individual differences in fraction congruency effects.

#### **Decimals**

As illustrated in Fig. 5e and f, congruency effects in simultaneous and sequential tasks were not correlated when measured with accuracy, *r*(154) = 0.01, *p* = .991. However, there was a significant but weak correlation in congruency effects in simultaneous and sequential tasks when measured with RT, *r*(154) = 0.24, *p* = .002. This correlation remained significant applying Benjamini–Hochberg correction, *p* = .012. These results indicate that simultaneous and sequential tasks may not equally capture individual differences in decimal congruency effects.

### How simultaneous and sequential stimulus presentation influences the distance and the congruency effects

We then conducted preregistered linear mixed models to extend the comparison and correlation analyses. These models allow us to account for additional factors in one comprehensive model, including (1) item-by-item performance and participant-level performance, (2) interactions across conditions, and (3) distance calculated as a continuous variable rather than categorical (i.e., near vs. far). In these models, we estimated the effect of task format, distance, and congruency on performance, while accounting for individual variability. In the analyses, we focused on the interaction terms, since they would reveal how distance and congruency effects depended on task format. Additionally, given that multiple models were tested, we interpret results based on effect sizes and confidence intervals rather than relying solely on *p*-values for inference. Results for the accuracy analyses are reported as odds ratios, while results for RT analyses are reported as raw coefficients, interpretable as RT in milliseconds.

Overall, the results corroborated differences in distance and congruency effects across task formats. Furthermore, the results indicated that the congruency effect varied as a function of distance and task format. These results align with the hypothesis that task format influences how multicomponent numbers are processed. We summarize our findings in Table 5, and describe them in detail in the following paragraphs. Post hoc analyses exploring the interaction effects are broadly described here and shown in detail in the Supplementary Information and OSF.

**Table 5:** Summary of interactions observed in the regression models

Interaction	Integers	Fractions	Decimals
**Distance × Task format**	✓	✓	✓
**Congruency × Task format**	✓	✓	✓
**Distance × Congruency × Task format**	✓	✓	✓

^✓^Indicates significant interaction with either accuracy or RT

#### **Integers**

As described in Table 6, the model with accuracy as the outcome variable indicated significant interactions between distance and task format, and congruency and task format. The two-way interaction between distance and congruency, and the three-way interaction between distance, congruency, and task format were not significant. Post hoc analyses (see Supplementary Information S4) indicated a stronger distance effect in the simultaneous task than in the sequential task. Additionally, the congruency effect was significant in the simultaneous task but only marginally significant (*p* = .046) in the sequential task.

**Table 6:** Linear mixed models

Predictor	Integers			Fractions			Decimals		
**Outcome = Accuracy**	**OR**	**95% CI**	***p***	**OR**	**95% CI**	***p***	**OR**	**95% CI**	***p***
Distance	1.69	1.28, 2.23	<.001	2.22	1.84, 2.68	<.001	1.16	1.00, 1.34	.050
Congruency (incongruent)	0.95	0.69, 1.31	.748	0.88	0.68, 1.14	.338	0.96	0.79, 1.17	.661
Task (simultaneous)	4.58	3.03, 6.91	<.001	1.93	1.54, 2.42	<.001	4.84	3.70, 6.33	<.001
Distance × congruency (incongruent)	1.01	0.72, 1.43	.936	1.45	1.11, 1.89	.006	1.05	0.86, 1.28	.619
Distance × task (simultaneous)	2.06	1.26, 3.35	.004	1.33	1.04, 1.70	.021	0.75	0.58, 0.98	.037
Congruency (incongruent) × task (simultaneous)	0.22	0.14, 0.36	<.001	0.58	0.43, 0.78	<.001	0.25	0.18, 0.34	<.001
Distance x congruency (incongruent) × task (simultaneous)	0.74	0.43, 1.26	.269	1.05	0.77, 1.45	.744	0.95	0.70, 1.31	.765
*AIC*	6,072.40			10,346.50			7,516.46		
**Outcome = RT**	**β**	**95% CI**	***p***	**β**	**95% CI**	***p***	**β**	**95% CI**	***p***
Distance	−50.03	−56.37, −43.68	<.001	−71.14	−81.06, −61.22	<.001	−16.60	−22.71, −10.49	<.001
Congruency (incongruent)	−12.70	−21.55, −3.84	.005	88.12	72.74, 103.49	<.001	−34.66	−44.10, −25.22	<.001
Task (simultaneous)	−34.14	−42.86, −24.41	<.001	249.10	236.37, 261.83	<.001	55.17	46.96, 63.37	<.001
Distance × congruency (incongruent)	−5.80	−14.87, 3.36	.210	−35.16	−49.05, −21.28	<.001	−4.70	−13.07, 3.67	.271
Distance × task (simultaneous)	6.65	−1.95, 15.24	.130	−38.80	−51.54, −26.06	<.001	2.26	−5.93, 10.46	.588
Congruency (incongruent) × task (simultaneous)	33.03	20.67, 45.39	<.001	29.03	10.82, 47.23	.002	34.75	23.12, 46.39	<.001
Distance x congruency (incongruent) × task (simultaneous)	−14.79	−27.20, −2.38	.019	21.28	3.15, 39.42	.021	24.02	12.40, 35.65	<.001
*AIC*	336,586.00			337,367.80			319,526.70		

*OR* odds ratio, *CI* confidence interval

The RT model showed a significant interaction between congruency and task format. There was a three-way interaction between distance, congruency, and task format. There was no two-way interaction between distance and congruency or between distance and task format. Post hoc analyses (see Supplementary Information S4) indicated that the congruency effect was significant in the simultaneous task but only marginally significant (*p* = .047) in the sequential task. Additionally, in the simultaneous task, the congruency effect became weaker as the distances between numbers increased. In the sequential task, the congruency effect was only marginally significant in near distances (*p* = .050) but became nonsignificant as the distances between numbers increased.

#### **Fractions**

As described in Table 6, the model with accuracy as the outcome variable indicated that there were significant interactions between distance and congruency, and distance and task format. There was also an interaction between congruency and task format. The three-way interaction between distance, congruency, and task was not significant. Post hoc analyses (see Supplementary Information S5) indicated a stronger congruency effect in near than far distances. Additionally, there was a stronger distance effect in the simultaneous than in the sequential task. Finally, there was a significant congruency effect in the simultaneous task but not in the sequential task.

The RT model revealed significant interactions between distance and congruency, distance and task format, and congruency and task format. There was also a three-way interaction between distance, congruency, and task. Post hoc analyses (see Supplementary Information S5) indicated a stronger congruency effect in near than far distances. Additionally, there was a stronger distance effect in the simultaneous than in the sequential task, as well as a stronger congruency effect in the simultaneous than the sequential task. Finally, there was a significant congruency effect in both near and far distances in the simultaneous task. However, in the sequential task, the congruency effect became weaker as distances increased.

#### **Decimals**

As described in Table 6, the model with accuracy as the outcome variable indicated that there were significant interactions between distance and task format, and congruency and task format. The two-way interaction between distance and congruency, and the three-way interaction between distance, congruency, and task were not significant. Post hoc analyses (see Supplementary Information S6) revealed a reversed distance effect (i.e., higher accuracy in near than far distances) in the simultaneous task, which contrasted with a standard distance effect in the sequential task (i.e., higher accuracy in far than near distances). There was a congruency effect in the simultaneous task, while the congruency effect was nonsignificant in the sequential task.

The RT model showed an interaction between congruency and task format. There was also a three-way interaction between distance, congruency, and task format. The two-way interactions between distance and congruency and between distance and task format were not significant. Post hoc analyses (see Supplementary Information S6) indicated a nonsignificant congruency effect in the simultaneous task and a reversed congruency effect (i.e., faster responses in incongruent than congruent trials) in the sequential task. When exploring the three-way interaction, we found that the congruency effect was reversed in near distances but standard (i.e., incongruent trials slower than congruent) in far distances in the simultaneous task. In contrast, the congruency effect was reversed (i.e., incongruent trials faster than congruent trials) in both near and far distances in the sequential task.

### Exploratory analyses

In the current study, we modeled congruency to grasp fragmented number processing of two-digit integer, fraction, and decimal numbers, but, an alternative way to investigate fragmented processing is by modeling participants’ performance based on the distance between numerical components. In the preregistration, we planned to contrast the role of holistic and fragmented distances by conducting linear mixed models. However, the distance between fragments of multicomponent numbers often correlates with holistic distance, which can give rise to multicollinearity issues. To account for this, we conducted an exploratory analysis using Lasso regression instead. Lasso regression identifies the most relevant predictors in a model by penalizing the coefficients of less predictive variables, shrinking them to zero. This statistical model can handle multicollinearity, being a good alternative for our analysis with holistic and componential distances.

We show detailed results in Supplementary Information (S7–S15). In models with either accuracy or RT in *integer comparisons* entered as outcome variables, and holistic distance (e.g., 32 vs. 47, holistic distance = 15), decade distance (e.g., **3**2 vs. **4**7, decade distance = 1), and unit distance (e.g., 3**2** vs. 4**7**, unit distance = 5) entered as predictors, only holistic distances were selected by the Lasso regressions.

In models with either accuracy or RT in *fraction comparisons* entered as outcome variables, and holistic distance (e.g., 1/8 vs. 8/9, holistic distance = 0.76), numerator distance (e.g., **1**/8 vs. **8**/9, numerator distance = 7), and denominator distance (e.g., 1/**8** vs. 8/**9**, denominator distance = 1) entered as predictors, holistic distances, numerator distances, and denominator distances were all selected by the Lasso models as significant predictors. However, the effects of numerator and denominator distances were weaker than the effects of holistic distance. Furthermore, denominator distances had an inverse association with participants’ performance. We also investigated how different strategies explained performance in the fraction comparison task. Previous studies have shown that, when comparing two fractions, participants may focus on the difference between numerator and denominator (gap strategy), or use a reverse-denominator strategy (i.e., larger denominator = smaller fraction; e.g., Gómez & Dartnell 2019; Gonzalez-Forte et al., 2020). Furthermore, considering different componential strategies, some previous studies have defined congruence in fraction tasks including a third category: neutral items, those with one component whose magnitude aligns with holistic magnitude, and another that conflicts with holistic magnitude (e.g., 6/7 vs. 3/8; Gómez & Dartnell, 2019; Morales et al., 2020). We show these exploratory analyses on OSF. Overall, they indicated that participants were not relying primarily on the gap or reverse-denominator strategy, and that a congruency effect was still observed across tasks, even when only including congruent and neutral items.

Finally, in models with accuracy and RT in *decimal comparisons* entered as outcome variables, and holistic distance (e.g., 0.3 vs. 0.57, holistic distance = 0.27), whole number distance (suggesting that participants were not integrating the digits as a decimal magnitude, but processing them individually as a whole number; e.g., 0.**3** vs. 0.**57**, whole number distance = 54), and tenths place distance (e.g., 0.**3** vs. 0.**5**7, tenths place distance, 2) entered as predictors, the Lasso models selected a mix of holistic distance and tenths place distance. In the simultaneous task, tenths place was the best predictor of accuracy, and holistic distance was the best predictor of RT. In the sequential task, holistic distance was the best predictor of accuracy, and the tenths place was the best predictor of RT. These results suggest that participants engaged in strategies compatible with holistic multicomponent number processing and a solid understanding of place value when solving our tasks, rather than solely a fragmented processing strategy.

## Discussion

In this study, we investigated how sequential and simultaneous stimulus presentation in 2AFC tasks influences holistic and fragmented information processing using multicomponent numbers. Whether information processing varies as a function of presentation format has been debated. Some findings indicate that people process information similarly in simultaneous and sequential 2AFC tasks (e.g., Moeller et al., 2013). Others suggest that people focus on isolated stimulus components in simultaneous 2AFC tasks, but integrate these components into a holistic representation in sequential 2AFC tasks (Ganor-Stern et al., 2009). Because multicomponent numbers have yielded mixed evidence for holistic and fragmented processing, they provide a solid test case for evaluating effects or stimulus presentation (e.g., Bonato et al., 2007; Dehaene et al., 1990; Nuerk et al., 2004; Toomarian & Hubbard, 2018). The inconsistencies among holistic and fragmented multicomponent numbers may arise, at least in part, from differences in how multicomponent number stimuli are presented in 2AFC tasks. Therefore, this study may also inform other comparisons of sequential versus simultaneous 2AFC paradigms with different stimuli.

To investigate how 2AFC task format influences multicomponent number processing, we examined how the distance and congruency effects—commonly interpreted as signatures of holistic and fragmented processing, respectively—vary across simultaneous and sequential two-digit integer, fraction, and decimal comparison tasks. Our first main finding was that participants showed distance and congruency effects across all multicomponent number types. This finding indicates that all types of multicomponent numbers were being processed both holistically and in a fragmented way, suggesting hybrid processing. The second main finding was that across multicomponent number types, the strength of the distance and congruency effects differed between simultaneous and sequential comparison tasks, suggesting that task format plays a critical role in shaping processing strategies. Therefore, our results indicate that the stimulus presentation in 2AFC influences strategies used in information processing. In the following paragraphs, we discuss these two main findings.

### Hybrid processing of multicomponent numbers

Previous studies have found that adults rely on both holistic and fragmented strategies when solving multicomponent number comparison tasks, suggesting hybrid processing (DeWolf & Vosniadou, 2015; Nuerk & Willmes, 2005; Van Hoof et al., 2020). Our findings align with this model. Participants’ responses for two-digit integers, fractions, and decimal comparisons displayed some degree of distance effect based on absolute numerical distance, suggesting that multicomponent numbers were processed holistically. There was also a congruency effect across all number types, suggesting fragmented processing of individual components. Together, these results support a form of hybrid processing.

Nuances in the type of hybrid processing emerged across different number types and across 2AFC task formats. Across all number types, we observed a significant interaction between distance, congruency, and task format, suggesting that distance and congruency may not be isolated from each other. Additionally, exploratory Lasso regression indicated that holistic distance was a robust predictor of participants’ performance in both simultaneous and sequential 2AFC tasks, even when some evidence for fragmented processing was observed. Together, these results suggest that participants do not process two-digit integers, fractions, and decimals solely in a holistic or fragmented way. Instead, they may engage in hybrid processing, dynamically shifting strategies in response to specific situational demands such as whether stimulus presentation is simultaneous or sequential in 2AFC tasks.

Flexibility in multicomponent number processing strategies likely extends beyond experimental tasks to everyday contexts. For example, during grocery shopping, people might compare numbers—such as $2.99 per pound versus $6.49 for a 2-pound bag—presented simultaneously when the products are displayed side by side on the same shelf, but sequentially when they appear in different parts of the aisle. Similarly, during online shopping, people might encounter multicomponent numbers presented simultaneously when comparing items within the same website, but sequentially when switching between websites. Given the different demands of these contexts, people may apply different strategies to compare multicomponent numbers depending on presentation format, including both holistic and fragmented processing.

Aligned with a dual-processing framework (Van Hoof et al., 2020), we argue that cognitive load may explain shifts between holistic and fragmented processing across numerical distances and between simultaneous and sequential 2AFC tasks. The dual-processing framework proposes that individuals rely on two cognitive systems to process information: system 1, which is fast, intuitive, and automatic, and system 2, which is slower, more effortful, and deliberate (Evans, 2008; Graziano, 2018; Van Hoof et al., 2020). When solving tasks, both systems can be activated simultaneously and may come into conflict, influencing which strategy is ultimately used.

If people concurrently engage in both holistic and fragmented multicomponent number processing, as proposed by the hybrid model of dual-digit processing (Nuerk & Willmes, 2005), these strategies may compete during number comparisons. Fragmented processing may be faster and more intuitive, whereas holistic processing may be slower and more deliberate. Holistic processing may be especially slow when comparing numbers that are close in magnitude (i.e., near-distance conditions), as these comparisons require greater cognitive effort. Slower holistic processing may give way to fragmented strategies, which could explain the stronger congruency effect we observed in near-distance conditions. In contrast, when numbers are separated by a far distance, holistic comparisons are less cognitively demanding and can be executed more quickly. As a result, holistic processing may dominate in far-distance conditions, reducing reliance on fragmented cues and thereby weakening the congruency effect.

### 2AFC task format influences multi-component number processing

Understanding how the reliability and validity of experimental measures, such as the 2AFC experimental paradigm, influence cognitive theories remains a challenge in psychology (Bach, 2024; Flake et al., 2022; Zorowitz & Niv, 2023). To explore the impact of 2AFC task format on information processing, we focused on how the distance and congruency effects varied across simultaneous and sequential multicomponent number 2AFC tasks.

Our results revealed distinct processing patterns for two-digit integers, fractions, and decimals related to the 2AFC task format. In the two-digit integer comparisons, we observed stronger distance and congruency effects in the simultaneous than in the sequential 2AFC task, especially when accounting for individual differences in our linear mixed model. We also found nonsignificant correlations between the distance and congruency effects across the two task formats. Consistent with findings from Ganor-Stern and colleagues (2009), these results suggest that participants engage different strategies depending on task format and, further, that simultaneous and sequential tasks may differentially capture individual differences in two-digit integer number processing.

In fraction comparisons, the distance and congruency effects were present in both task formats but were stronger in the simultaneous task. These findings suggest that participants are capable of processing the holistic magnitude of fractions, but their judgments are also influenced by the magnitudes of the numerator and denominator. While both task formats captured individual variability, the simultaneous task appeared more sensitive to capturing interference effects from component-based processing strategies, similar to findings from two-digit integers.

In decimal comparisons, we observed greater divergence between the simultaneous and sequential tasks than in two-digit integer and fraction comparisons. In accuracy data, we observed a significant distance effect in the sequential task (i.e., less accurate for *near* trials) but a reversed distance effect in the simultaneous task (i.e., less accurate for *far* trials). In contrast, there was a congruency effect in the sequential task but no effect in the simultaneous task. In RT analyses, the congruency effect was reversed in the sequential task across all distances and shifted from a standard to a reversed effect in the simultaneous task as numerical distances decreased from far to near. These contrasting patterns suggest that participants engage different processing strategies when comparing decimals in simultaneous versus sequential formats, potentially reflecting distinct underlying cognitive mechanisms. In particular, the sequential task may have encouraged more holistic processing of decimals, or strategies compatible with place-value understanding, whereas the simultaneous task may have elicited more fragmented strategies.

A reversed congruency effect in our sequential decimal comparison task, and in the near-distance trials of the simultaneous task in our RT analyses, partially replicates findings from Rosenberg-Lee and colleagues (2023). They reported a standard congruency effect in simultaneous decimal comparisons when analyzing accuracy, but a reversed effect in analyses with RT. While speculative, one possible explanation for the reversed congruency effect in decimal comparisons is that it reflects specific characteristics of the item set used in the task. In the item set used by Rosenberg-Lee and colleagues (2023), which we adopted in this study, congruent trials often included pairs with the same digit in the tenths place, whereas incongruent trials did not. If participants relied on the tenths place to make their decisions (as suggested by our Lasso regression results), the lack of variability in that digit for congruent trials may have encouraged them to integrate the magnitude expressed by the hundredths place to resolve ambiguity, slowing down their responses relative to incongruent trials. It is also important to note that congruent trials included very small decimal numbers (e.g., 0.1), while incongruent trials included very large decimal numbers (e.g., 0.9), which may also have impacted number processing strategies. Future studies should investigate whether these differences in stimulus structure influence participants' strategies, including potential speed–accuracy trade-offs, that may contribute to slower RT in congruent than incongruent trials in sequential 2AFC tasks.

Altogether, these findings indicate that stimulus presentation in 2AFC tasks influences information processing. These results corroborate previous findings showing that participants engage in distinct strategies when solving simultaneous versus sequential 2AFC comparison tasks (Ganor-Stern et al., 2009; Zhang & Wang, 2005), with simultaneous tasks eliciting more fragmented strategies and sequential tasks eliciting more holistic strategies.

One potential explanation for this finding is that the presentation format in 2AFC tasks may modulate a competition between holistic and fragmented processing. In simultaneous tasks, both stimuli are presented at once, increasing the likelihood that attention is split not only between the two holistic stimuli (e.g., 43 vs. 27) but also between their individual components (e.g., 4, 3, 2, 7). Under strict time constraints, this division of attentional resources—combined with the need to make a rapid decision—may exacerbate reliance on intuitive, automatic fragmented processing. In contrast, since sequential tasks present only one stimulus at a time, they may reduce attentional demands. We argue that when participants are presented with only one stimulus at a time (e.g., “43” shown on a screen and “27” shown on the following screen), their attention is distributed across fewer elements (e.g., 43, 4, and 3 on the first screen), and the inter-stimulus interval may provide them with sufficient time for more deliberate processing. When the second stimulus appears, participants may be more likely to engage in comparison based on holistic information. This reduced competition for attentional resources, along with lower time pressure, may support holistic processing and reduce the congruency effect in sequential 2AFC tasks relative to simultaneous tasks.

### Implications for cognitive studies using 2AFC tasks in non-numerical domains

Our findings indicate that simultaneous and sequential 2AFC tasks elicit different strategies when people are processing complex, multicomponent stimuli. While our results have been constrained to numerical stimuli, they align with findings from previous studies showing that 2AFC task format influences information processing in perceptual domains. For instance, using factor analysis, Mou and colleagues (2024) showed that simultaneous and sequential nonsymbolic magnitude comparisons load into different factors, suggesting that they measure distinct latent constructs. Furthermore, Larsen and colleagues (1999) have shown differences across task formats in visual processing. While participants tend to create a mental model of a visual shape presented first, and then compare it with a second shape in sequential tasks, they tend to engage in a series of transformations based on details of the shapes when they are presented simultaneously. Thus, consistent with previous findings (e.g., Ganor-Stern et al., 2009; Larsen et al., 1999; Mou et al., 2024; Zhang & Wang, 2005), our results suggest that simultaneous and sequential 2AFC tasks should not be used interchangeably in cognitive research.

Future research should examine the psychometric properties of 2AFC tasks in greater detail. For instance, it is important to assess their construct validity using methods such as factor analysis. In simultaneous tasks, stimuli are presented on the same screen, requiring participants to divide their attention across two numbers. In sequential tasks, because the stimuli are shown on different screens, participants must retain the first stimulus in memory to compare it with the second. Due to these intrinsic differences, the two formats may engage distinct cognitive processes—for example, inhibitory control in simultaneous tasks and working memory in sequential tasks. Future studies should investigate how simultaneous and sequential tasks tap into these other constructs, and statistically control for working memory and inhibitory control, or at least account for them in the overall processing model.

### Limitations and future directions

A main limitation of the current study is that it remains unclear how its findings translate to real-world contexts, when people encounter stimuli in different presentation formats and solve problems without externally determined time pressures. Thus, the current findings do not explain whether the observed effects reflect characteristics of the 2AFC paradigm itself or broader principles of information processing. Future studies should investigate whether people engage in holistic, fragmented, or hybrid processing when solving problems with stimuli presented either simultaneously or sequentially across more naturalistic contexts to extend the external validity of the current findings.

It is also unclear how our findings may have been influenced by the specific comparison pairs used across experimental paradigms. We adapted comparison pairs from prior peer-reviewed research to account for distance and congruency effects (Binzak & Hubbard, 2020; DeWolf et al., 2016; Nuerk et al., 2001; Rosenberg-Lee et al., 2023). This approach allowed us to build on established paradigms and ensured that the stimuli had been tested for suitability in similar experimental contexts. However, it remains uncertain how our results would be replicated when holistic distance, fragmented distance, and congruency are manipulated differently. For example, our fraction comparison list included pairs with shared numerators and denominators, which may encourage fragmented processing (Toomarian & Hubbard, 2018). Similarly, our decimal comparison list included pairs with identical tenths digits, which may influence participants’ strategies. Future research should systematically investigate how item-level characteristics contribute to variability in performance across simultaneous and sequential 2AFC tasks.

Another factor that may have influenced the observed differences across simultaneous and sequential 2AFC tasks is variation in the temporal parameters of the tasks, including interstimulus and intertrial intervals. In the sequential 2AFC tasks, participants had more time to process each stimulus compared to the simultaneous tasks. Thus, the interactions among distance, congruency, and task format could reflect the time available for processing rather than an intrinsic effect of 2AFC task format. Future studies should examine how holistic and fragmented processing strategies vary across simultaneous and sequential 2AFC tasks when interstimulus and intertrial intervals are manipulated (e.g., by increasing or decreasing time pressure). Such investigations will provide a more robust understanding of how temporal parameters influence performance across different types of 2AFC tasks.

Finally, another key limitation of the current study is that our sample consisted of undergraduate students. As such, participants had extensive training in mathematics and were likely highly proficient with multicomponent numbers. Participants with higher proficiency may be familiar with a broader range of comparison strategies and may flexibly select among them depending on 2AFC task format. In contrast, individuals with lower proficiency and a limited strategy repertoire may show similar distance and congruency effects across simultaneous and sequential tasks. Future research should investigate whether the observed differences between simultaneous and sequential 2AFC tasks in a highly educated sample are replicated in populations with lower educational attainment (e.g., adults with low literacy) and at other developmental stages (e.g., children and adolescents).

## Conclusion

Taken together, our findings suggest that simultaneous and sequential 2AFC tasks should not be used interchangeably in cognitive research. In particular, we found that the 2AFC stimulus presentation format influenced the distance and congruency effects in two-digit integer, fraction, and decimal tasks. Our results suggest that, rather than relying exclusively on holistic or fragmented processing strategies, participants may shift dynamically between the two, depending on task format. A critical implication of this finding is that future studies using 2AFC tasks to investigate information processing should carefully consider whether simultaneous or sequential formats are adequate for their research questions, and interpret their findings in light of task features.

## Supplementary Information

Below is the link to the electronic supplementary material.

Supplementary file1 (DOCX 603 KB)

## Data Availability

The aggregated dataset used in this study, along with preregistration of the main research questions and research materials, is shared via our OSF page (https://osf.io/yzgws/overview?view_only=ca841e5901a74320a3e2145ee5b1d37e).

## Code availability

The analysis code (R markdown) and supplementary analyses are shared via our OSF page (https://osf.io/yzgws/overview?view_only=ca841e5901a74320a3e2145ee5b1d37e).
