# Supplementary material for: Congruency and distance effects vary across simultaneous and sequential two-digit integer, fraction, and decimal 2AFC tasks
Source: Behav Res Methods. 2026 Apr 13;58(5):117. doi: 10.3758/s13428-026-02958-6 (PMC13076428; doi:10.3758/s13428-026-02958-6)
Supplement: Supplementary file 1 — Supplementary file1 (DOCX 603 KB) [file 13428_2026_2958_MOESM1_ESM.docx]

**Congruency and distance effects vary across simultaneous and sequential two-digit integer, fraction, and decimal 2AFC tasks**

**Supplementary Information**

S1. Table: Stimuli List - Integer Comparisons

S2. Table: Stimuli List - Fraction Comparisons

S3. Table: Stimuli List - Decimal Comparisons

S4. Figure: Interaction Analyses - Integer Comparisons

S5. Figure: Interaction Analyses - Fraction Comparisons

S6. Figure: Interaction Analyses - Decimal Comparisons

S7. Table: Lasso Regression - Holistic and fragmented distances as predictors of performance in integer number comparisons

S8. Table: Lasso Regression - Holistic and fragmented distances as predictors of performance in fraction comparisons

S9.Table: Lasso Regression - Holistic and fragmented distances as predictors of performance in decimal comparisons

S10. Figure: Holistic and fragmented distances as predictors of performance in simultaneous integer number comparisons

S11. Figure: Holistic and fragmented distances as predictors of performance in sequential integer number comparisons

S12. Figure: Holistic and fragmented distances as predictors of performance in simultaneous fraction comparisons

S13. Figure: Holistic and fragmented distances as predictors of performance in sequential fraction comparisons

S14. Figure: Holistic and fragmented distances as predictors of performance in simultaneous decimal comparisons

S15. Figure: Holistic and fragmented distances as predictors of performance in sequential decimal comparisons

**S1. Stimuli List - Integer Comparisons**

| **Item** | **Pair** | **Congruency** | **Distance** | **Distance**  **Category** | **Item** | **Pair** | **Congruency** | **Distance** | **Distance**  **Category** |
| --- | --- | --- | --- | --- | --- | --- | --- | --- | --- |
| **01** | 31 vs. 46 | Congruent | 15 | Near | **01** | 46 vs. 51 | Incongruent | 5 | Near |
| **02** | 32 vs. 47 | Congruent | 15 | Near | **02** | 59 vs. 64 | Incongruent | 5 | Near |
| **03** | 41 vs. 56 | Congruent | 15 | Near | **03** | 68 vs. 73 | Incongruent | 5 | Near |
| **04** | 43 vs. 58 | Congruent | 15 | Near | **04** | 39 vs. 45 | Incongruent | 6 | Near |
| **05** | 52 vs. 67 | Congruent | 15 | Near | **05** | 46 vs. 52 | Incongruent | 6 | Near |
| **06** | 34 vs. 58 | Congruent | 24 | Near | **06** | 49 vs. 61 | Incongruent | 12 | Near |
| **07** | 45 vs. 69 | Congruent | 24 | Near | **07** | 38 vs. 52 | Incongruent | 14 | Near |
| **08** | 21 vs. 46 | Congruent | 25 | Near | **08** | 48 vs. 62 | Incongruent | 14 | Near |
| **09** | 34 vs. 59 | Congruent | 25 | Near | **09** | 39 vs. 54 | Incongruent | 15 | Near |
| **10** | 51 vs. 76 | Congruent | 25 | Near | **10** | 58 vs. 73 | Incongruent | 15 | Near |
| **11** | 25 vs. 79 | Congruent | 54 | Far | **11** | 28 vs. 71 | Incongruent | 43 | Far |
| **12** | 32 vs. 87 | Congruent | 55 | Far | **12** | 29 vs. 73 | Incongruent | 44 | Far |
| **13** | 34 vs. 89 | Congruent | 55 | Far | **13** | 46 vs. 91 | Incongruent | 45 | Far |
| **14** | 31 vs. 87 | Congruent | 56 | Far | **14** | 48 vs. 93 | Incongruent | 45 | Far |
| **15** | 21 vs. 78 | Congruent | 57 | Far | **15** | 25 vs. 71 | Incongruent | 46 | Far |
| **16** | 32 vs. 96 | Congruent | 64 | Far | **16** | 29 vs. 81 | Incongruent | 52 | Far |
| **17** | 24 vs. 89 | Congruent | 65 | Far | **17** | 38 vs. 91 | Incongruent | 53 | Far |
| **18** | 32 vs. 97 | Congruent | 65 | Far | **18** | 37 vs. 91 | Incongruent | 54 | Far |
| **19** | 21 vs. 87 | Congruent | 66 | Far | **19** | 26 vs. 81 | Incongruent | 55 | Far |
| **20** | 31 vs. 98 | Congruent | 67 | Far | **20** | 36 vs. 92 | Incongruent | 56 | Far |

**S2. Stimuli List - Fraction Comparisons**

| **Item** | **Pair** | **Congruency** | **Distance** | **Distance**  **Category** | **Item** | **Pair** | **Congruency** | **Distance** | **Distance**  **Category** |
| --- | --- | --- | --- | --- | --- | --- | --- | --- | --- |
| **01** | 3/4 vs. 6/7 | Congruent | 0.11 | Near | **01** | 1/4 vs. 1/7 | Incongruent | 0.11 | Near |
| **02** | 4/9 vs. 5/9 | Congruent | 0.11 | Near | **02** | 1/3 vs. 2/9 | Incongruent | 0.11 | Near |
| **03** | 8/9 vs. 7/9 | Congruent | 0.11 | Near | **03** | 2/5 vs. 2/7 | Incongruent | 0.11 | Near |
| **04** | 7/8 vs. 3/4 | Congruent | 0.13 | Near | **04** | 1/2 vs. 3/8 | Incongruent | 0.13 | Near |
| **05** | 6/7 vs. 5/7 | Congruent | 0.14 | Near | **05** | 1/9 vs. 1/4 | Incongruent | 0.14 | Near |
| **06** | 7/9 vs. 5/8 | Congruent | 0.15 | Near | **06** | 3/8 vs. 2/9 | Incongruent | 0.15 | Near |
| **07** | 7/8 vs. 5/7 | Congruent | 0.16 | Near | **07** | 2/7 vs. 1/8 | Incongruent | 0.16 | Near |
| **08** | 5/7 vs. 8/9 | Congruent | 0.17 | Near | **08** | 2/7 vs. 1/9 | Incongruent | 0.17 | Near |
| **09** | 3/8 vs. 5/9 | Congruent | 0.18 | Near | **09** | 4/9 vs. 5/8 | Incongruent | 0.18 | Near |
| **10** | 3/5 vs. 5/6 | Congruent | 0.23 | Near | **10** | 1/6 vs. 2/5 | Incongruent | 0.23 | Near |
| **11** | 5/9 vs. 2/9 | Congruent | 0.33 | Far | **11** | 1/2 vs. 1/6 | Incongruent | 0.33 | Far |
| **12** | 8/9 vs. 4/9 | Congruent | 0.44 | Far | **12** | 2/9 vs. 2/3 | Incongruent | 0.44 | Far |
| **13** | 6/7 vs. 2/5 | Congruent | 0.46 | Far | **13** | 6/7 vs. 3/8 | Incongruent | 0.48 | Far |
| **14** | 2/5 vs. 8/9 | Congruent | 0.49 | Far | **14** | 1/6 vs. 2/3 | Incongruent | 0.49 | Far |
| **15** | 8/9 vs. 3/8 | Congruent | 0.51 | Far | **15** | 5/8 vs. 1/9 | Incongruent | 0.51 | Far |
| **16** | 1/6 vs. 7/9 | Congruent | 0.61 | Far | **16** | 2/9 vs. 5/6 | Incongruent | 0.61 | Far |
| **17** | 7/8 vs. 1/4 | Congruent | 0.63 | Far | **17** | 6/7 vs. 2/9 | Incongruent | 0.63 | Far |
| **18** | 7/9 vs. 1/8 | Congruent | 0.65 | Far | **18** | 7/8 vs. 2/9 | Incongruent | 0.65 | Far |
| **19** | 7/8 vs. 1/7 | Congruent | 0.73 | Far | **19** | 1/8 vs. 6/7 | Incongruent | 0.73 | Far |
| **20** | 1/8 vs. 8/9 | Congruent | 0.76 | Far | **20** | 1/9 vs. 7/8 | Incongruent | 0.76 | Far |

**S3. Stimuli List - Decimal Comparisons**

| **Item** | **Pair** | **Congruency** | **Distance** | **Distance**  **Category** | **Item** | **Pair** | **Congruency** | **Distance** | **Distance**  **Category** |
| --- | --- | --- | --- | --- | --- | --- | --- | --- | --- |
| **01** | 0.1 vs. 0.19 | Congruent | 0.09 | Near | **01** | 0.3 vs. 0.21 | Incongruent | 0.09 | Near |
| **02** | 0.1 vs. 0.190 | Congruent | 0.09 | Near | **02** | 0.3 vs. 0.210 | Incongruent | 0.09 | Near |
| **03** | 0.2 vs. 0.29 | Congruent | 0.09 | Near | **03** | 0.4 vs. 0.31 | Incongruent | 0.09 | Near |
| **04** | 0.2 vs. 0.290 | Congruent | 0.09 | Near | **04** | 0.4 vs. 0.310 | Incongruent | 0.09 | Near |
| **05** | 0.3 vs. 0.39 | Congruent | 0.09 | Near | **05** | 0.5 vs. 0.41 | Incongruent | 0.09 | Near |
| **06** | 0.3 vs. 0.390 | Congruent | 0.09 | Near | **06** | 0.5 vs. 0.410 | Incongruent | 0.09 | Near |
| **07** | 0.6 vs. 0.69 | Congruent | 0.09 | Near | **07** | 0.8 vs. 0.71 | Incongruent | 0.09 | Near |
| **08** | 0.6 vs. 0.690 | Congruent | 0.09 | Near | **08** | 0.8 vs. 0.710 | Incongruent | 0.09 | Near |
| **09** | 0.7 vs. 0.79 | Congruent | 0.09 | Near | **09** | 0.9 vs. 0.81 | Incongruent | 0.09 | Near |
| **10** | 0.7 vs. 0.790 | Congruent | 0.09 | Near | **10** | 0.9 vs. 0.810 | Incongruent | 0.09 | Near |
| **11** | 0.5 vs. 0.68 | Congruent | 0.18 | Far | **11** | 0.9 vs. 0.72 | Incongruent | 0.18 | Far |
| **12** | 0.5 vs. 0.680 | Congruent | 0.18 | Far | **12** | 0.9 vs. 0.720 | Incongruent | 0.18 | Far |
| **13** | 0.1 vs. 0.37 | Congruent | 0.27 | Far | **13** | 0.7 vs. 0.43 | Incongruent | 0.27 | Far |
| **14** | 0.1 vs. 0.370 | Congruent | 0.27 | Far | **14** | 0.7 vs. 0.430 | Incongruent | 0.27 | Far |
| **15** | 0.2 vs. 0.47 | Congruent | 0.27 | Far | **15** | 0.8 vs. 0.53 | Incongruent | 0.27 | Far |
| **16** | 0.2 vs. 0.470 | Congruent | 0.27 | Far | **16** | 0.8 vs. 0.530 | Incongruent | 0.27 | Far |
| **17** | 0.3 vs. 0.57 | Congruent | 0.27 | Far | **17** | 0.9 vs. 0.63 | Incongruent | 0.27 | Far |
| **18** | 0.3 vs. 0.570 | Congruent | 0.27 | Far | **18** | 0.9 vs. 0.630 | Incongruent | 0.27 | Far |
| **19** | 0.1 vs. 0.46 | Congruent | 0.36 | Far | **19** | 0.9 vs. 0.54 | Incongruent | 0.36 | Far |
| **20** | 0.1 vs. 0.460 | Congruent | 0.36 | Far | **20** | 0.9 vs. 0.540 | Incongruent | 0.36 | Far |

**S4. Interaction Analyses - Integer Comparisons**

**
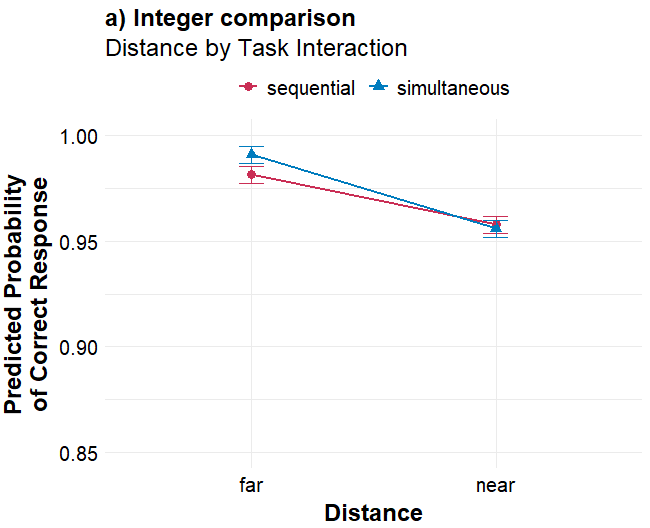

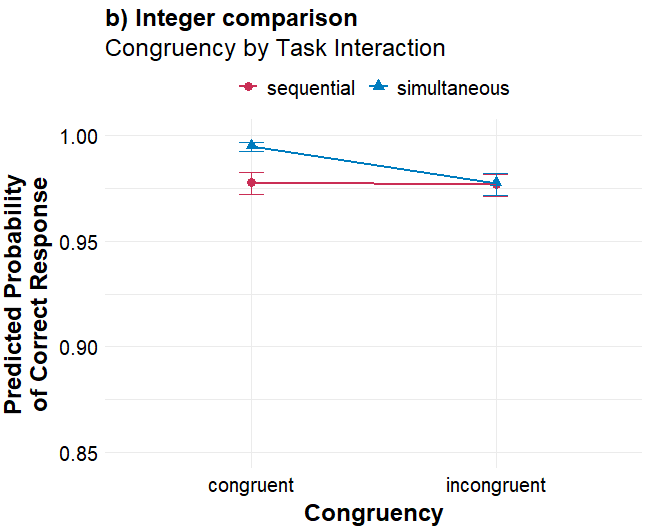
**

**
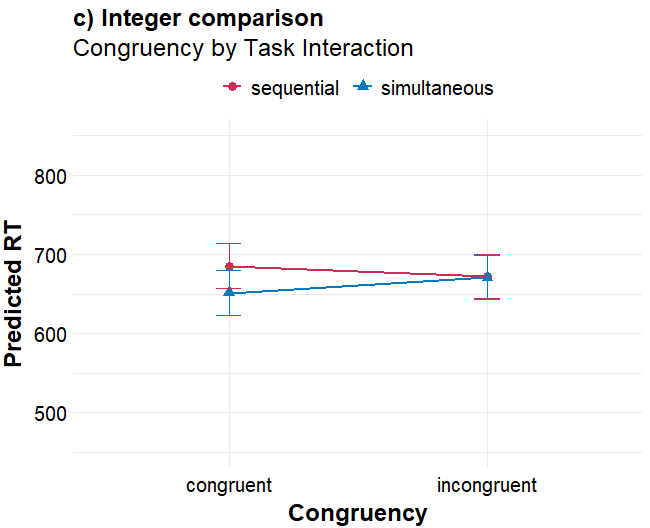

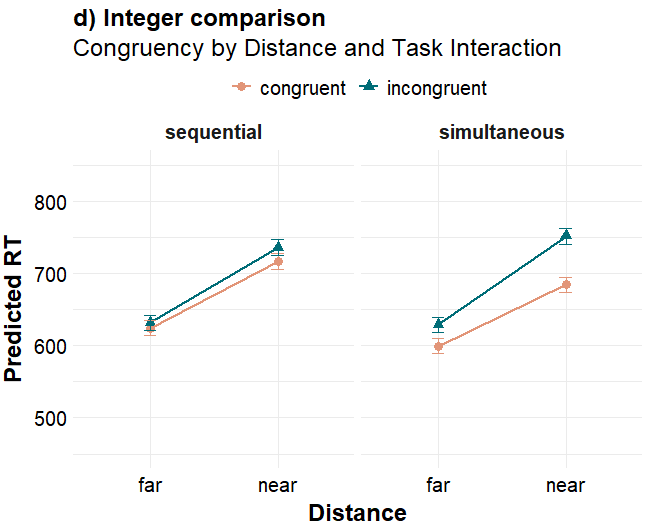
**

*Note.* We tested the interactions with distance entered as a continuous variable. However, we plot it as binary for easier interpretation. Results from the simple slopes analysis can be found on OSF: https://osf.io/yzgws/?view_only=c70ca02cdc0f4600b2ee4d5a602701f9

**S5. Interaction Analyses - Fraction Comparisons**

**
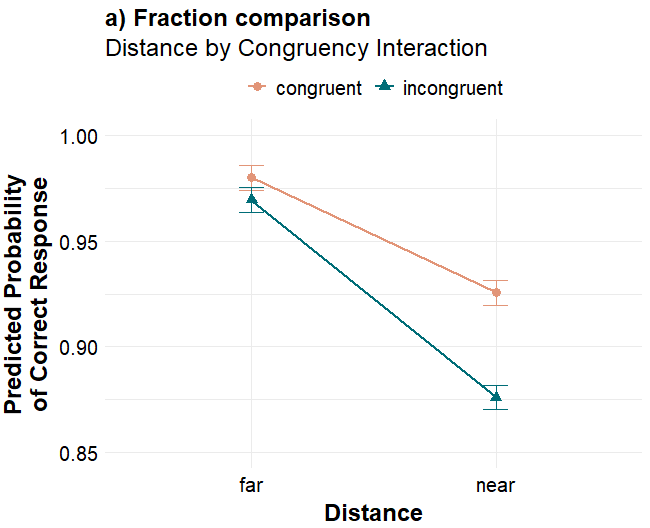

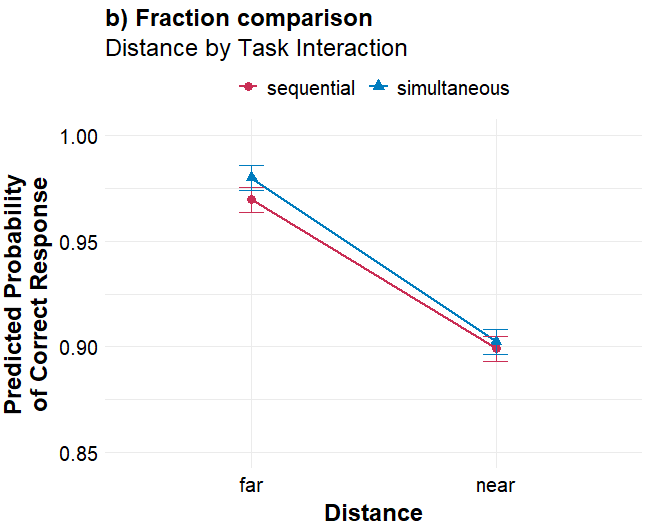

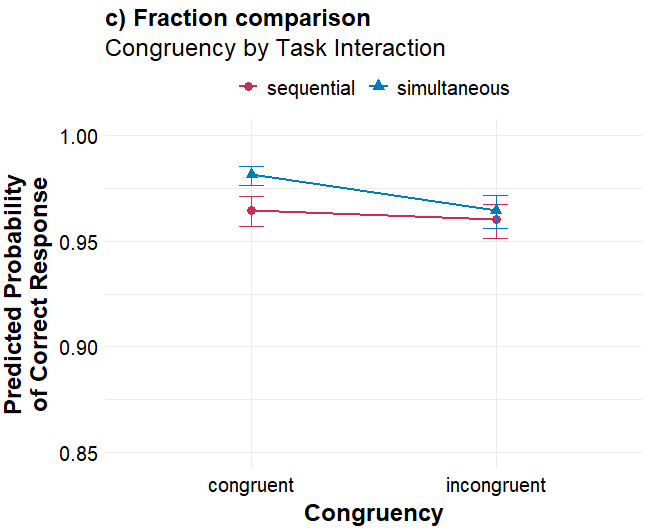

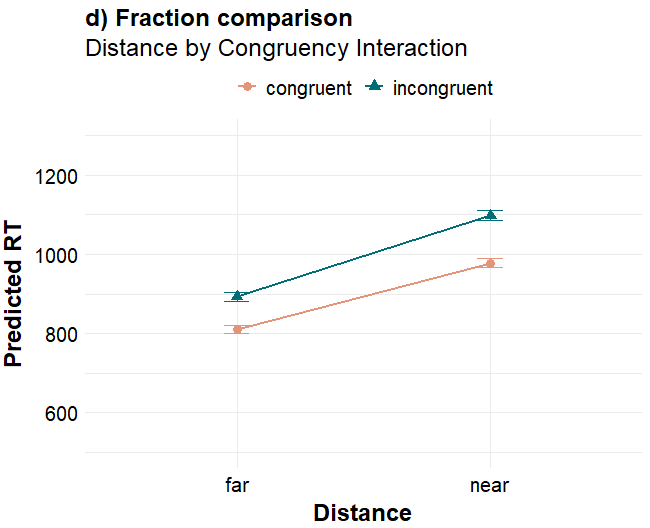
**

**
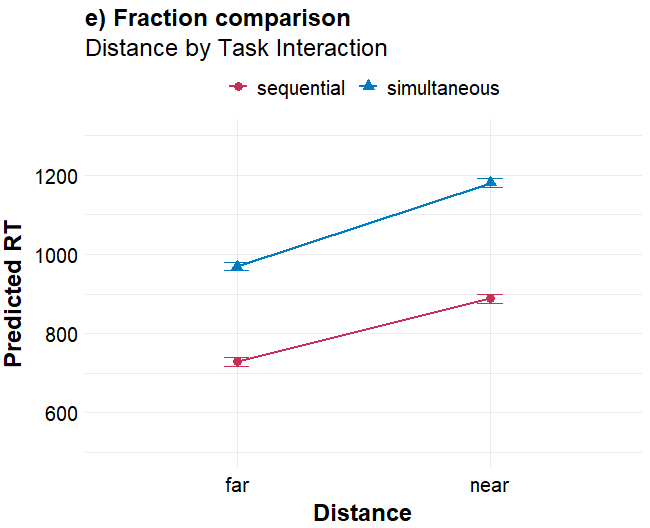

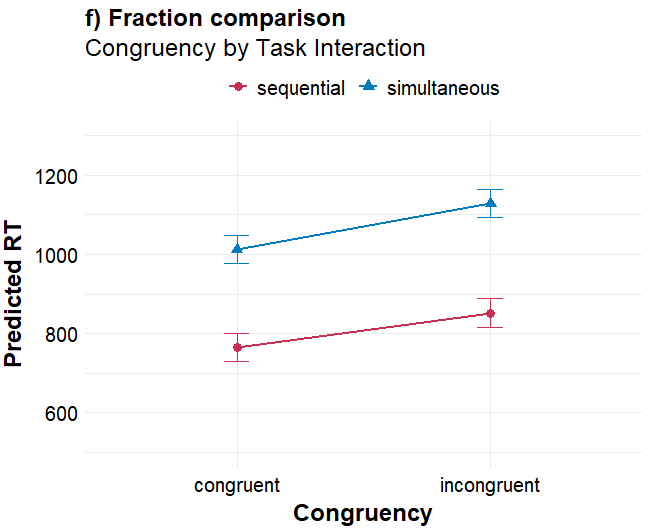
**

**
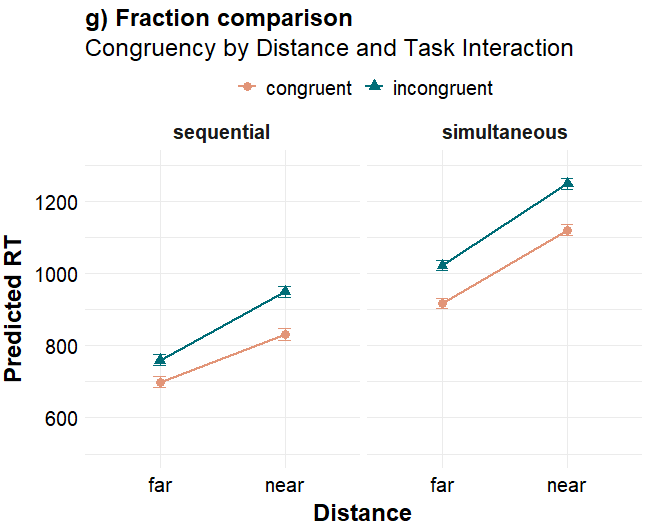
**

*Note.* We tested the interactions with distance entered as a continuous variable. However, we plot it as binary for easier interpretation. Results from the simple slopes analysis can be found on OSF: https://osf.io/yzgws/?view_only=c70ca02cdc0f4600b2ee4d5a602701f9

**S6. Interaction Analyses - Decimal Comparisons**

**
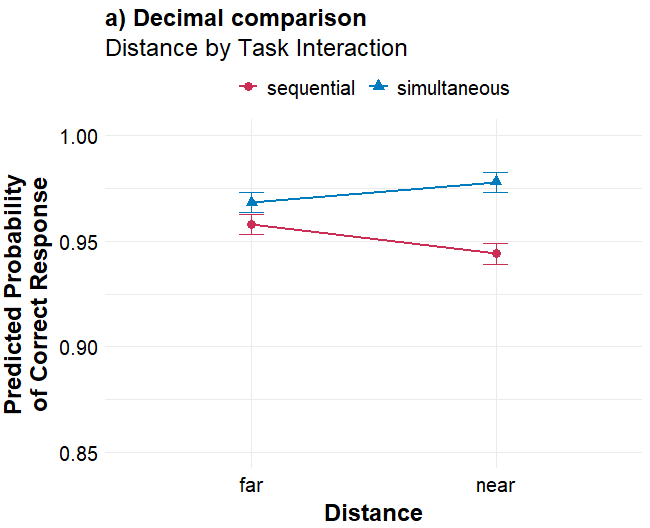

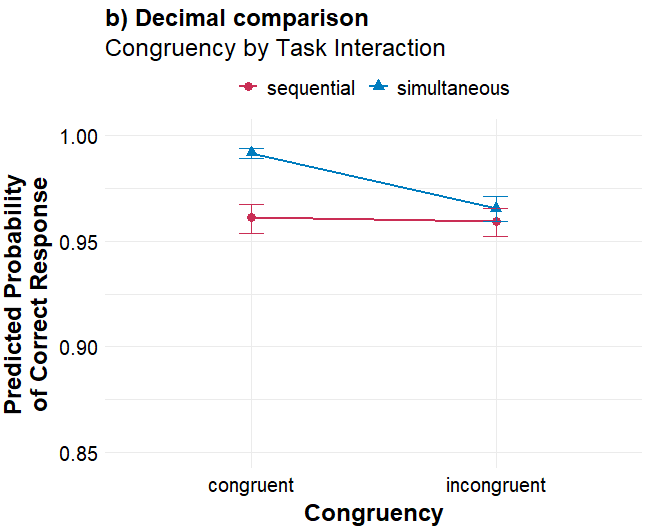
**

**
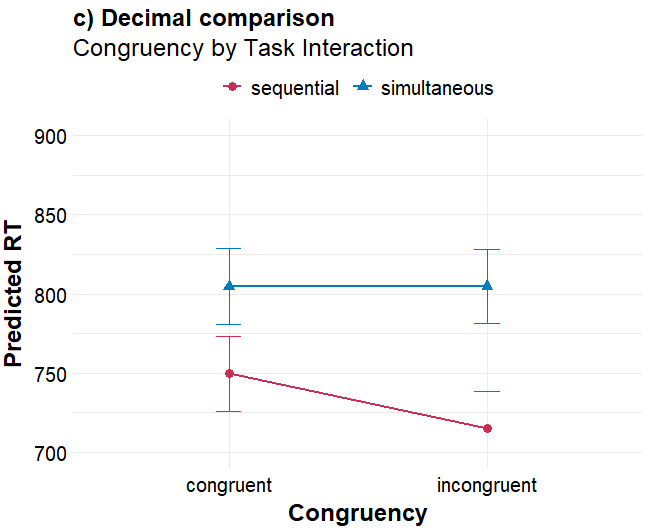

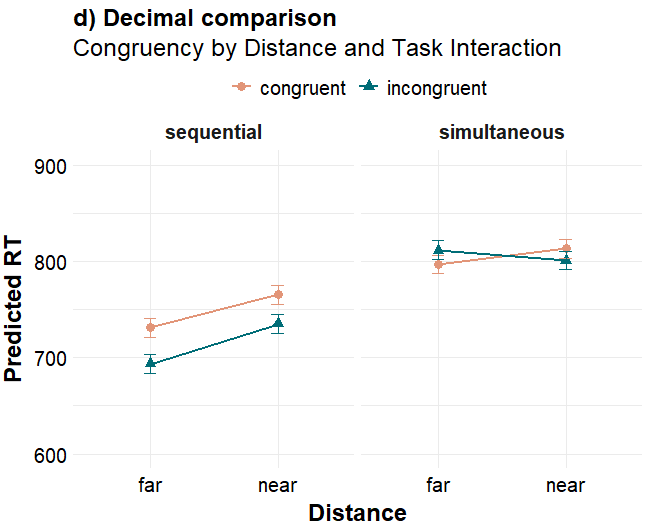
**

*Note.* We tested the interactions with distance entered as a continuous variable. However, we plot it as binary for easier interpretation. Results from the simple slopes analysis can be found on OSF: https://osf.io/yzgws/?view_only=c70ca02cdc0f4600b2ee4d5a602701f9

**S7. Lasso Regression: Holistic and fragmented distances as predictors of performance in integer number comparisons**

| **Task** | **Predictor** | **Coefficient** | **Selected** |
| --- | --- | --- | --- |
| **Simultaneous** | **Accuracy** | | |
|  | Integer distance | 0.001 | Yes |
|  | Decade distance | 0.000 | No |
|  | Unit distance | 0.000 | No |
|  | **RT** | | |
|  | Integer distance | 0.999 | Yes |
|  | Decade distance | 0.000 | No |
|  | Unit distance | 0.000 | No |
| **Sequential** | **Accuracy** | | |
|  | Integer distance | 0.0002 | Yes |
|  | Decade distance | 0.0000 | No |
|  | Unit distance | 0.0000 | No |
|  | **RT** | | |
|  | Integer distance | 0.999 | Yes |
|  | Decade distance | 0.000 | No |
|  | Unit distance | 0.000 | No |

**S8. Lasso Regression: Holistic and fragmented distances as predictors of performance in fraction comparisons**

| **Task** | **Predictor** | **Coefficient** | **Selected** |
| --- | --- | --- | --- |
| **Simultaneous** | **Accuracy** | | |
|  | Holistic distance | 0.120 | Yes |
|  | Numerator distance | 0.000 | No |
|  | Denominator distance | -0.029 | Yes |
|  | **RT** | | |
|  | Holistic distance | -198.63 | Yes |
|  | Numerator distance | -41.59 | Yes |
|  | Denominator distance | 12.53 | Yes |
| **Sequential** | **Accuracy** | | |
|  | Holistic distance | 0.119 | Yes |
|  | Numerator distance | 0.000 | No |
|  | Denominator distance | -0.008 | Yes |
|  | **RT** | | |
|  | Holistic distance | -176.78 | Yes |
|  | Numerator distance | -26.63 | Yes |
|  | Denominator distance | 10.89 | Yes |

**S9. Lasso Regression: Holistic and fragmented distances as predictors of performance in decimal comparisons**

| **Task** | **Predictor** | **Coefficient** | **Selected** |
| --- | --- | --- | --- |
| **Simultaneous** | **Accuracy** | | |
|  | Decimal distance | 0.000 | No |
|  | Tenth distance | -0.004 | Yes |
|  | Whole number distance | 0.000 | No |
|  | **RT** | | |
|  | Decimal distance | -42.93 | Yes |
|  | Tenth distance | 0.000 | No |
|  | Whole number distance | 0.042 | Yes |
| **Sequential** | **Accuracy** | | |
|  | Decimal distance | 0.0001 | Yes |
|  | Tenth distance | 0.000 | No |
|  | Whole number distance | 0.000 | No |
|  | **RT** | | |
|  | Decimal distance | 0.000 | No |
|  | Tenth distance | -15.81 | Yes |
|  | Whole number distance | 0.000 | No |

**S10. Holistic and fragmented distances as predictors of performance in simultaneous integer number comparisons**


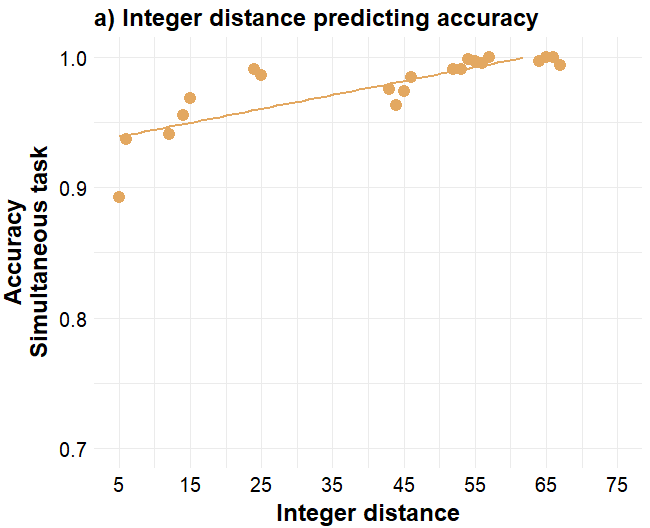

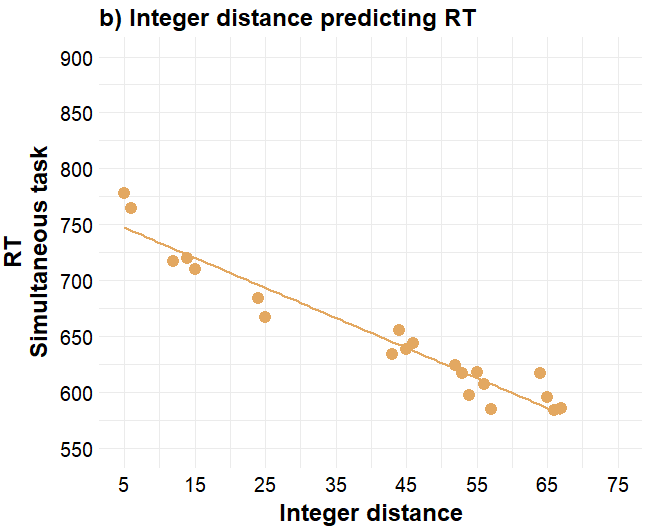


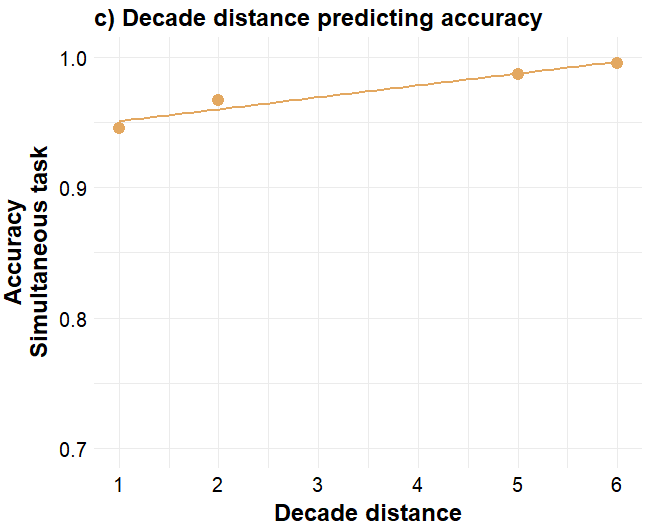

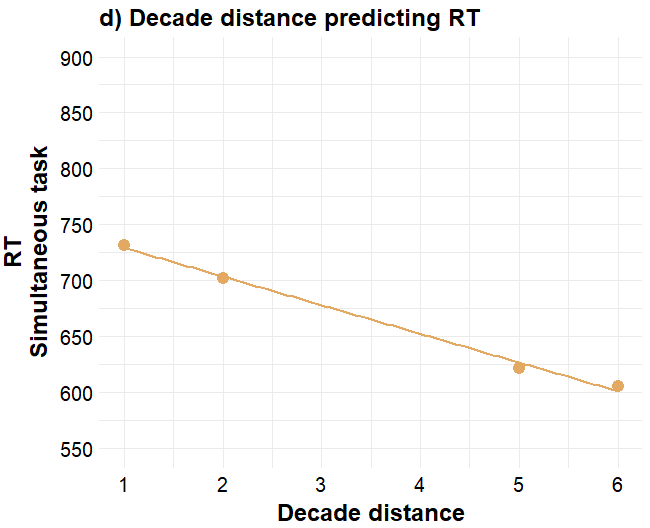


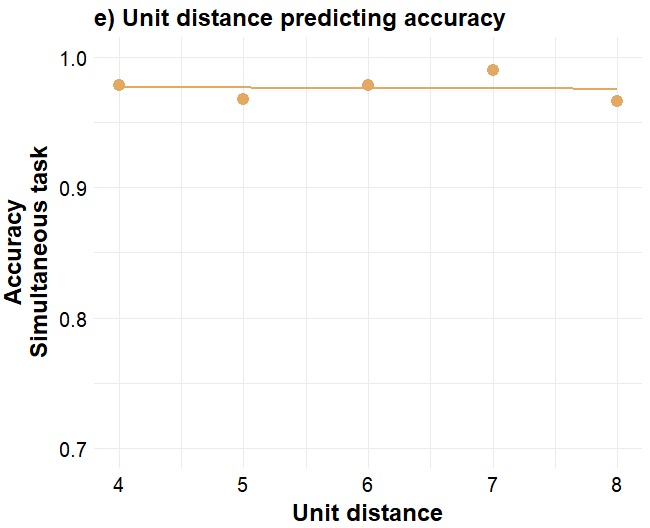

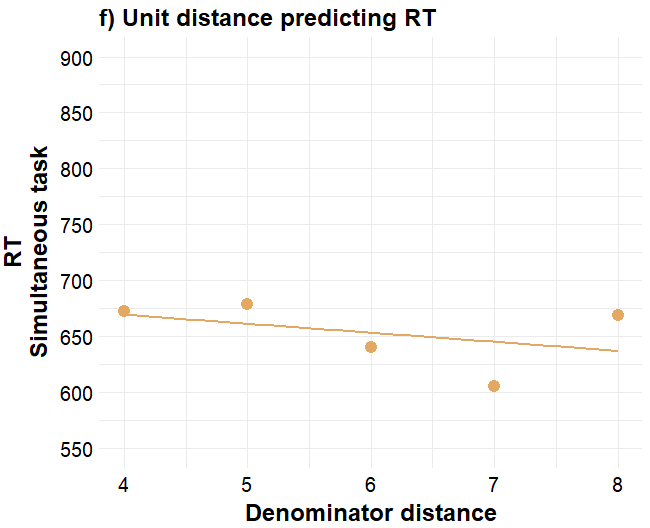


**S11. Holistic and fragmented distances as predictors of performance in sequential integer number comparisons**

**
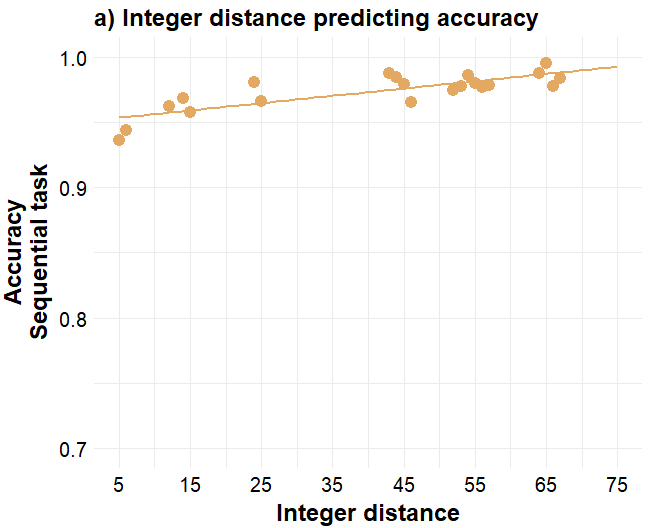

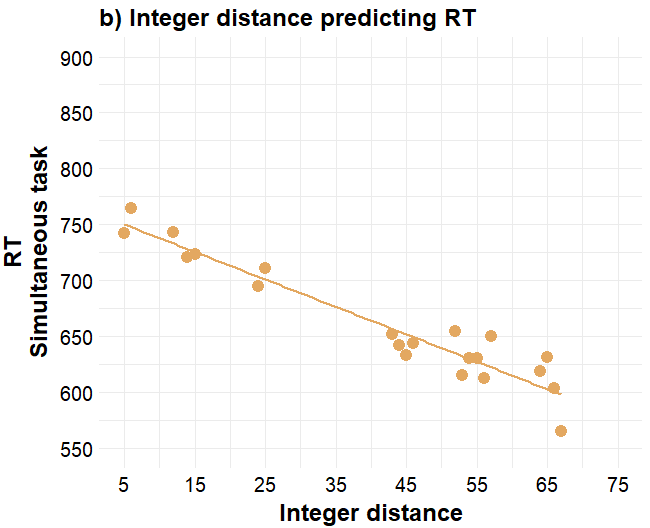
**

**
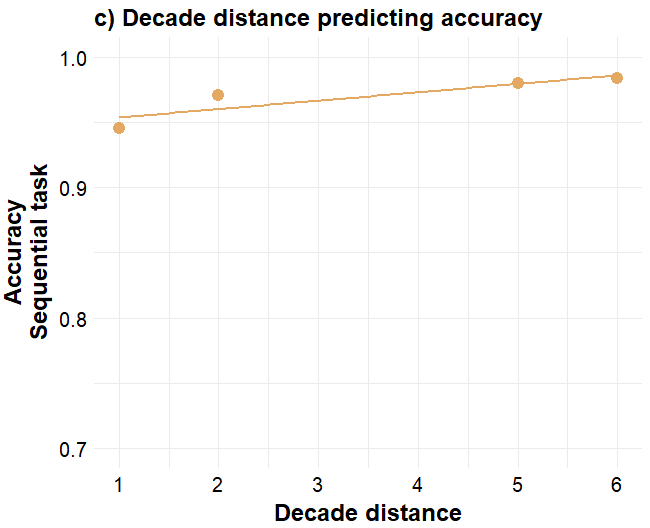

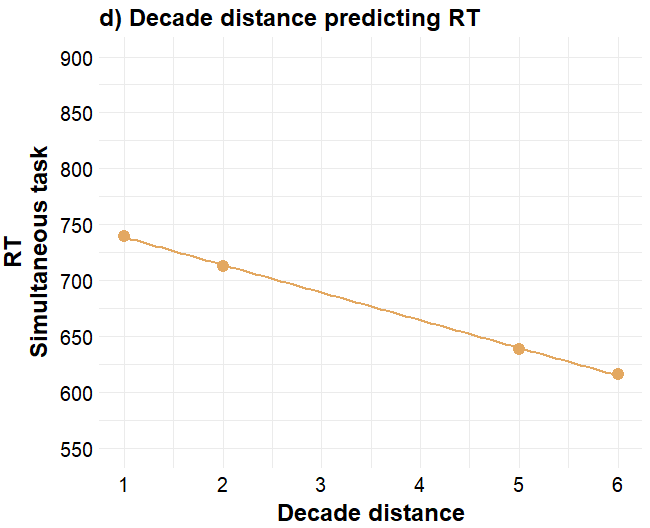
**

**
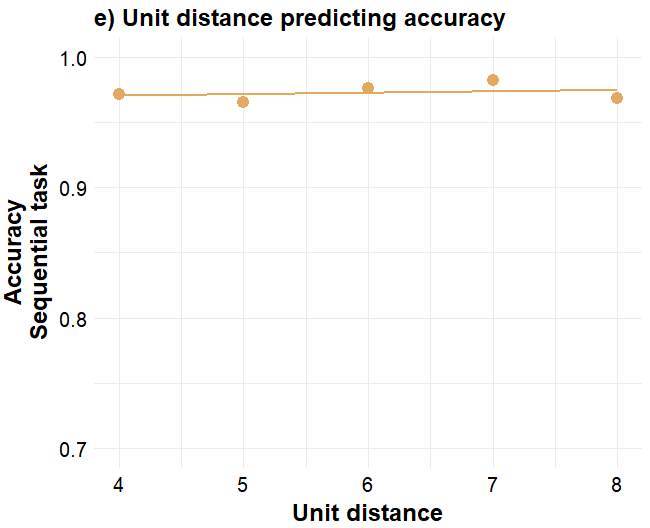

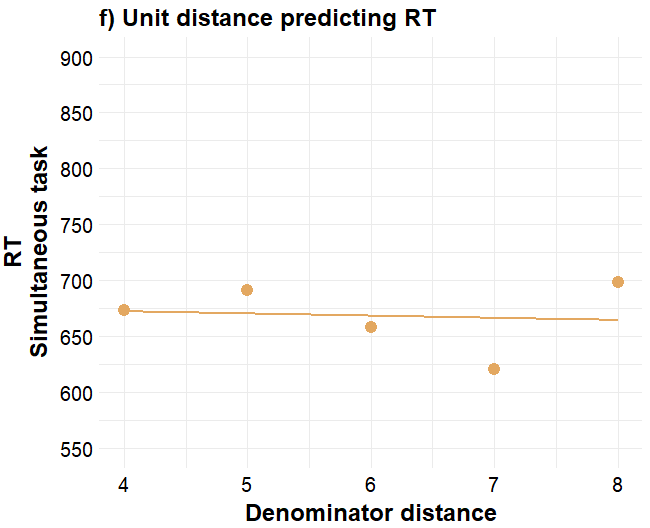
**

**S12. Holistic and fragmented distances as predictors of performance in simultaneous fraction comparisons**


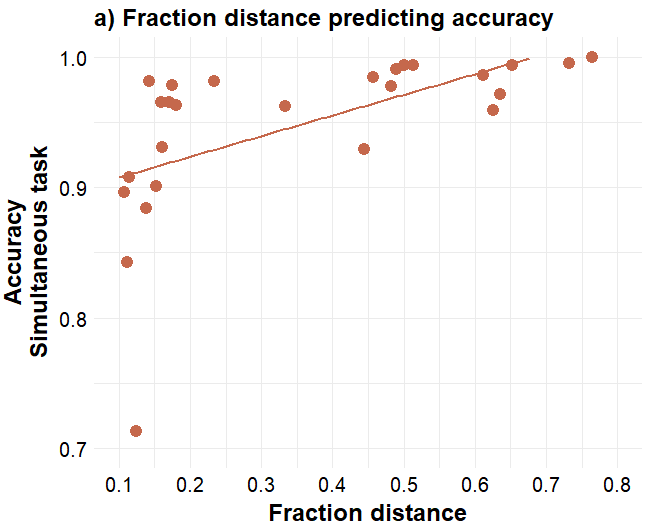

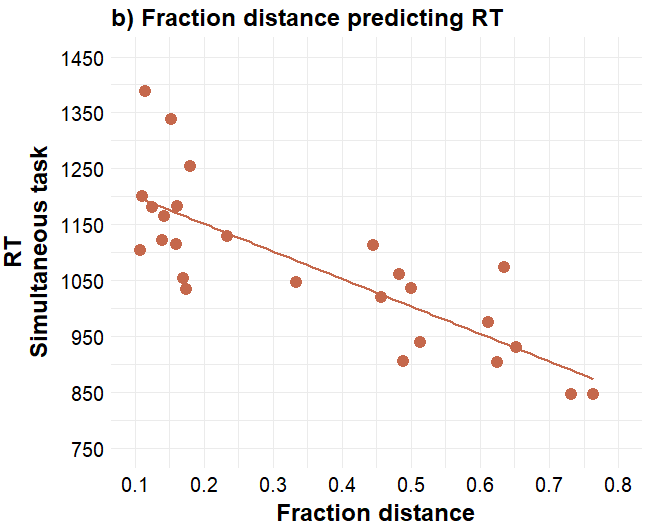


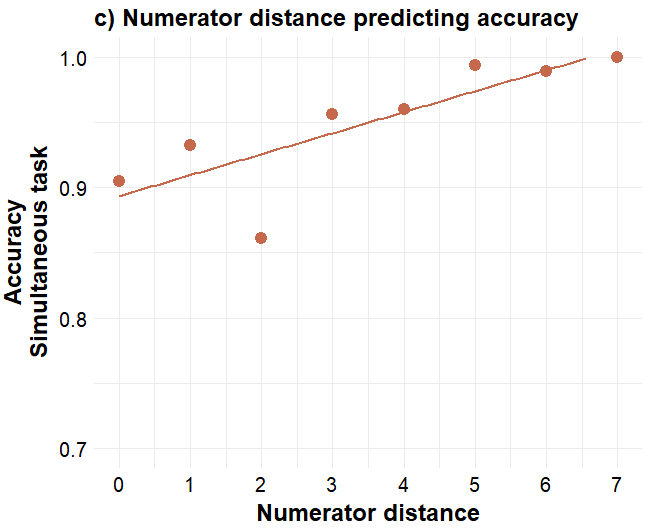

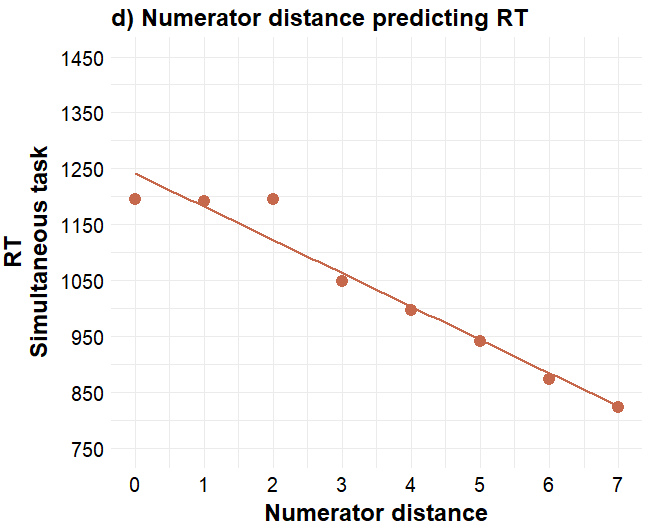


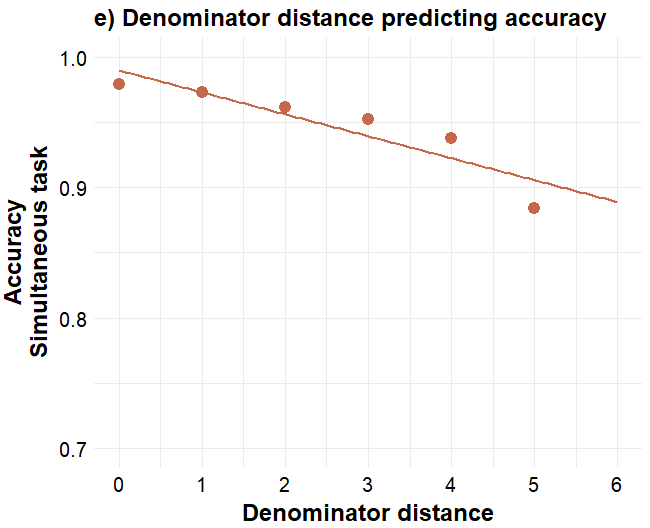

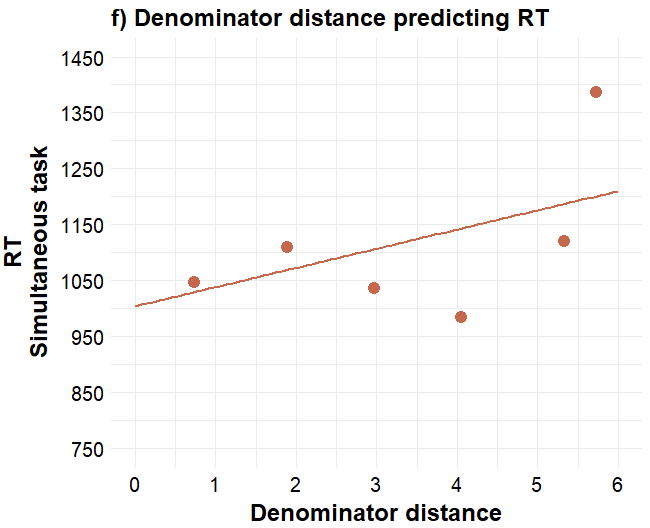


**S13. Holistic and fragmented distances as predictors of performance in sequential fraction comparisons**

**
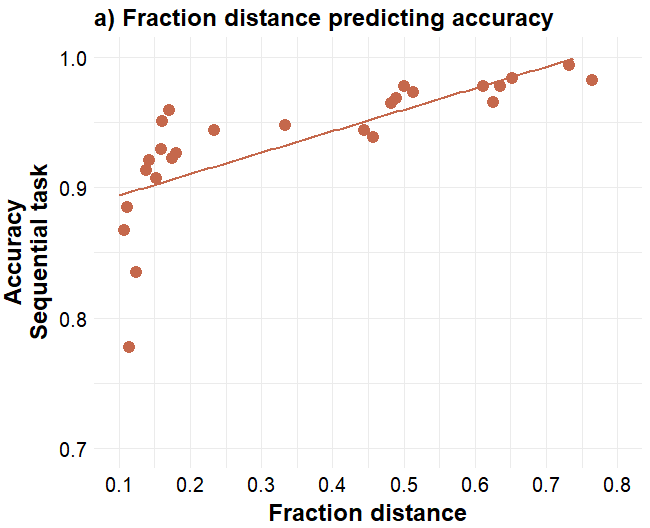

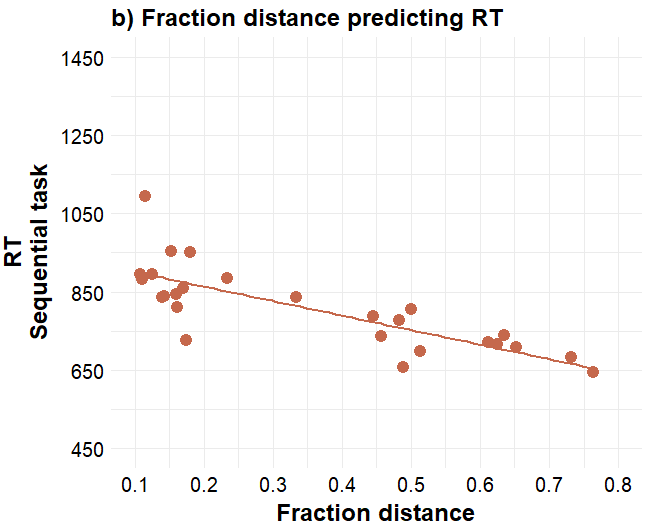
**

**
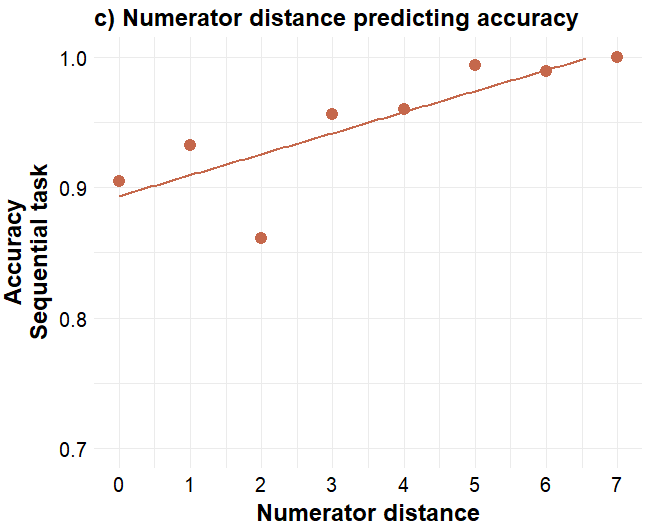

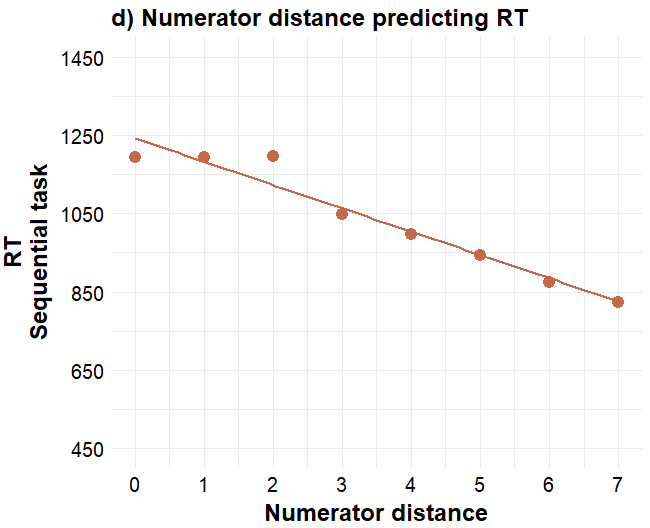
**

**
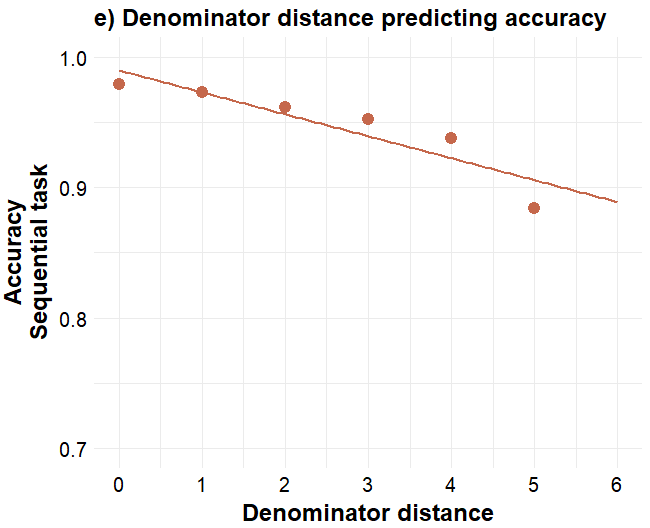

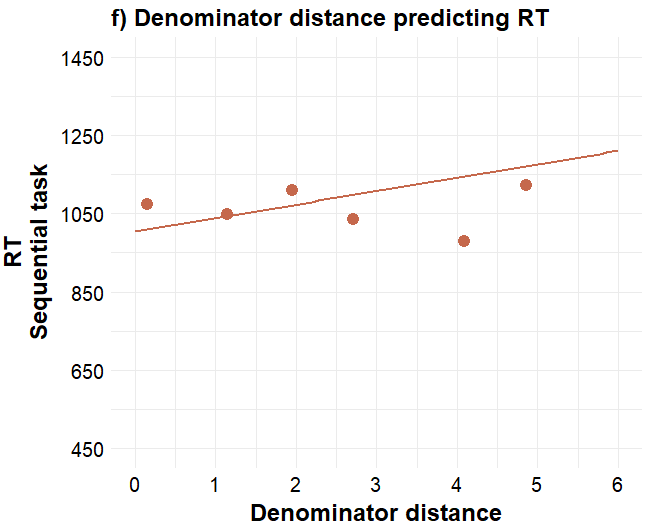
**

**S14. Holistic and fragmented distances as predictors of performance in simultaneous decimal comparisons**


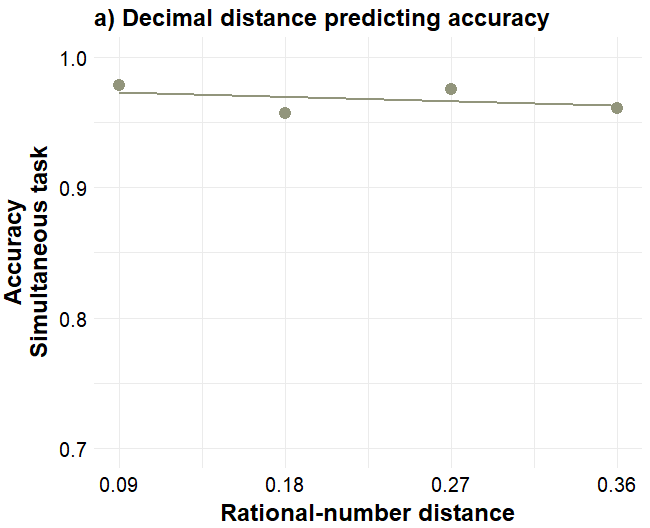

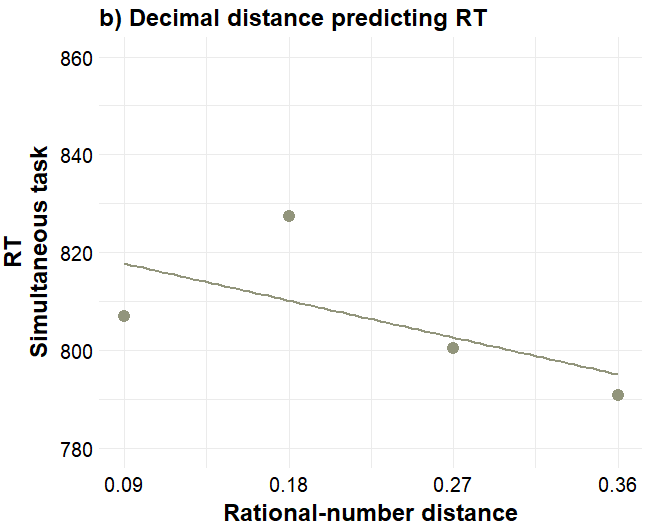


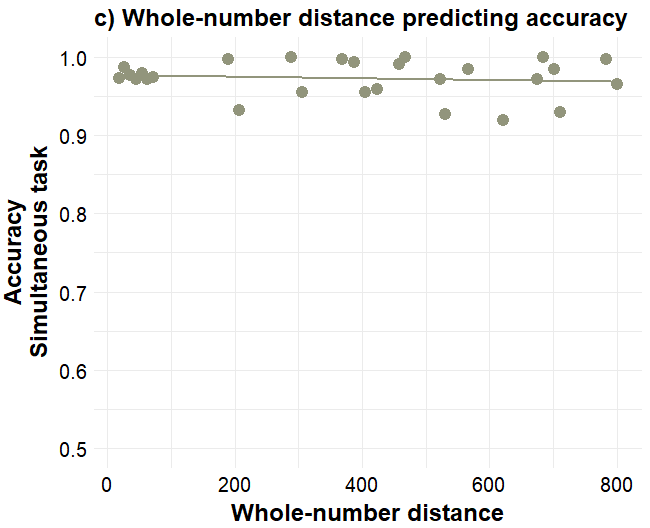

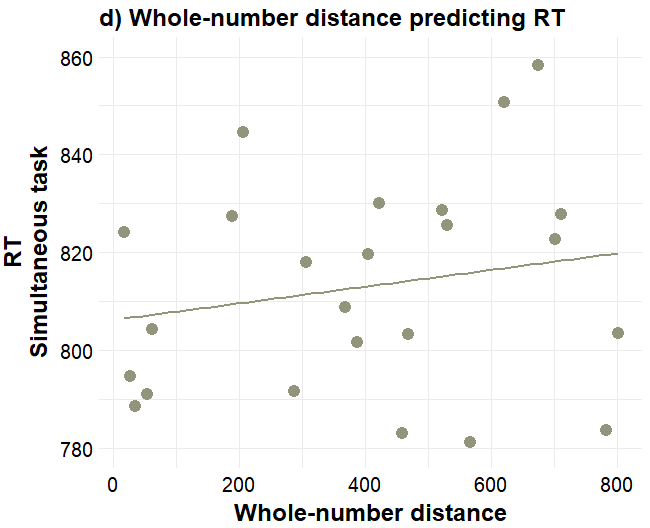


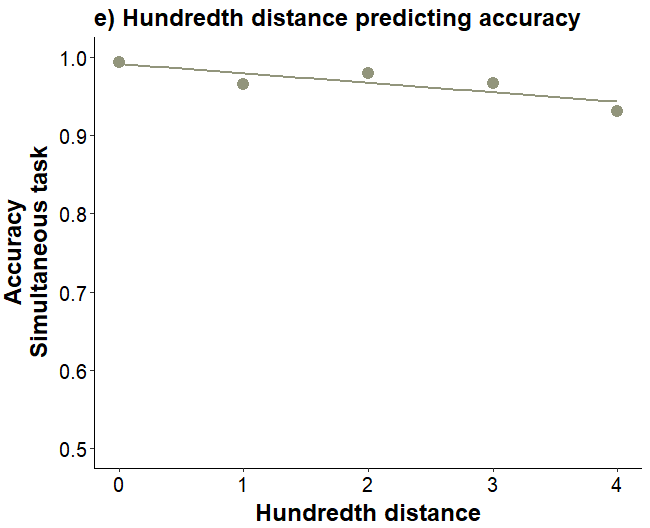

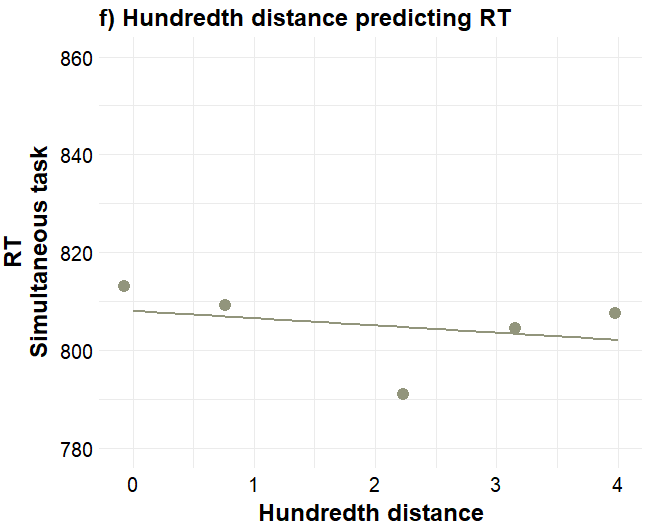


**S15. Holistic and fragmented distances as predictors of performance in sequential decimal comparisons**

**
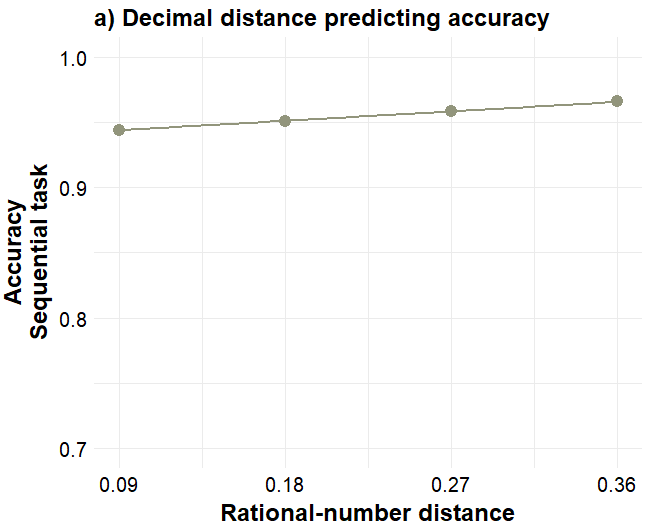

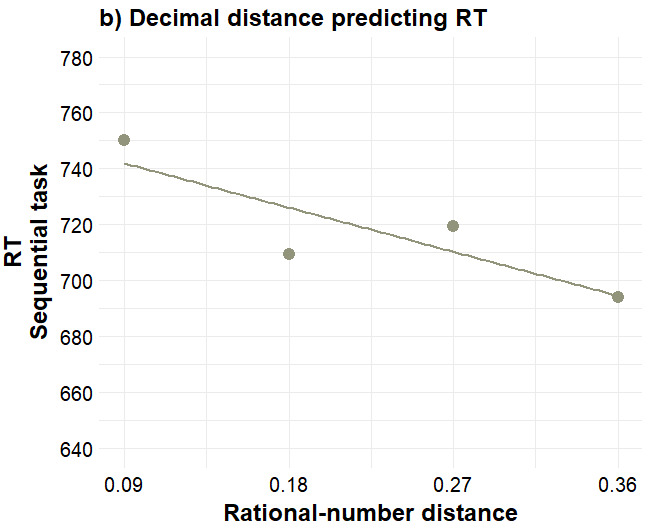

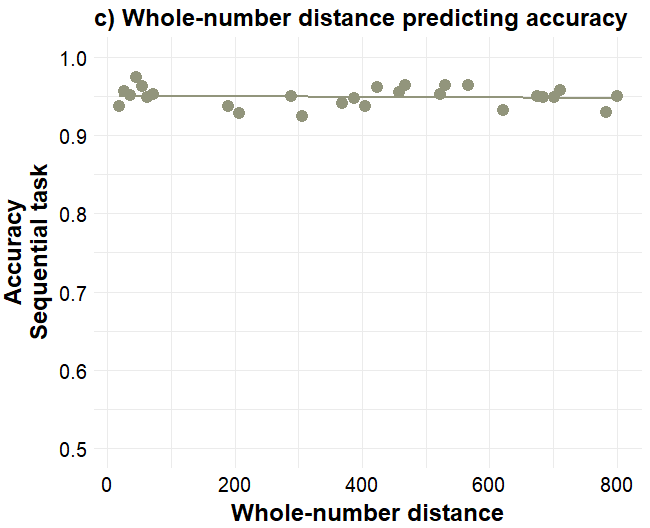

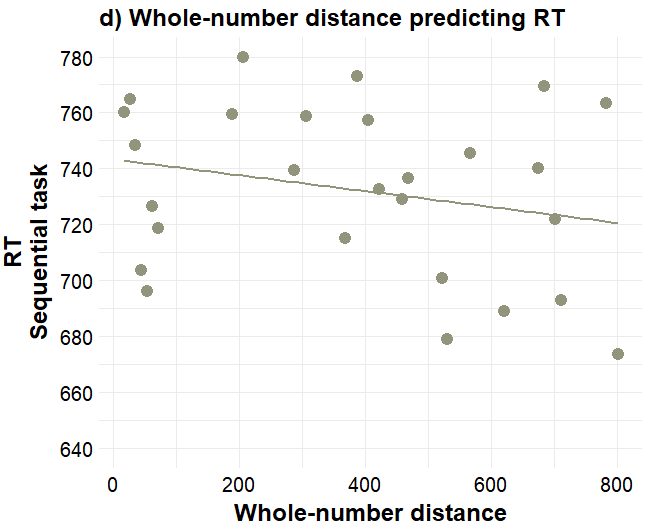
**

**
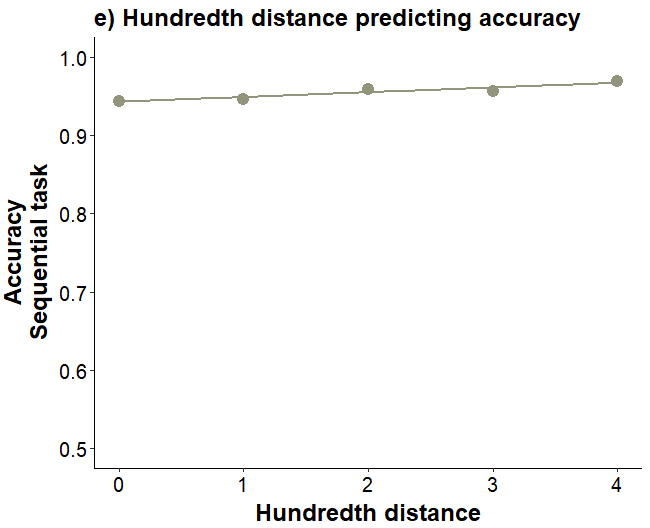

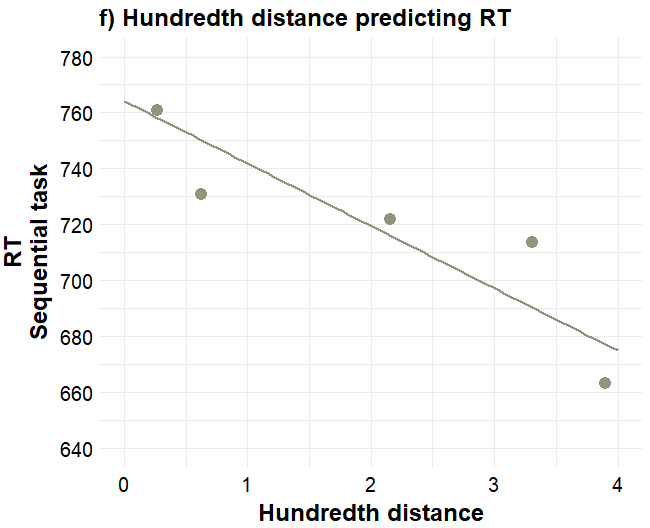
**
